# Supplementary material for: Bimodal endocytic probe for three-dimensional correlative light and electron microscopy
Source: Cell Rep Methods. 2022 May 16;2(5):100220. doi: 10.1016/j.crmeth.2022.100220 (PMC9142762; doi:10.1016/j.crmeth.2022.100220)
Supplement: Document S1. Figures S1–S4 [file mmc1.pdf]

Cell Reports Methods, Volume *2*

Supplemental information

Bimodal endocytic probe for three-dimensional correlative light and electron microscopy

Job Fermie, Leanne de Jager, Helen E. Foster, Tineke Veenendaal, Cecilia de Heus, Suzanne van Dijk, Corlinda ten Brink, Viola Oorschot, Lin Yang, Wei Li, Wally H. Müller, Stuart Howes, Andrew P. Carter, Friedrich Förster, George Posthuma, Hans C. Gerritsen, Judith Klumperman, and Nalan Liv

Supplemental Figures

Bimodal Endocytic Probe for Three-Dimensional Correlative Light and Electron Microscopy

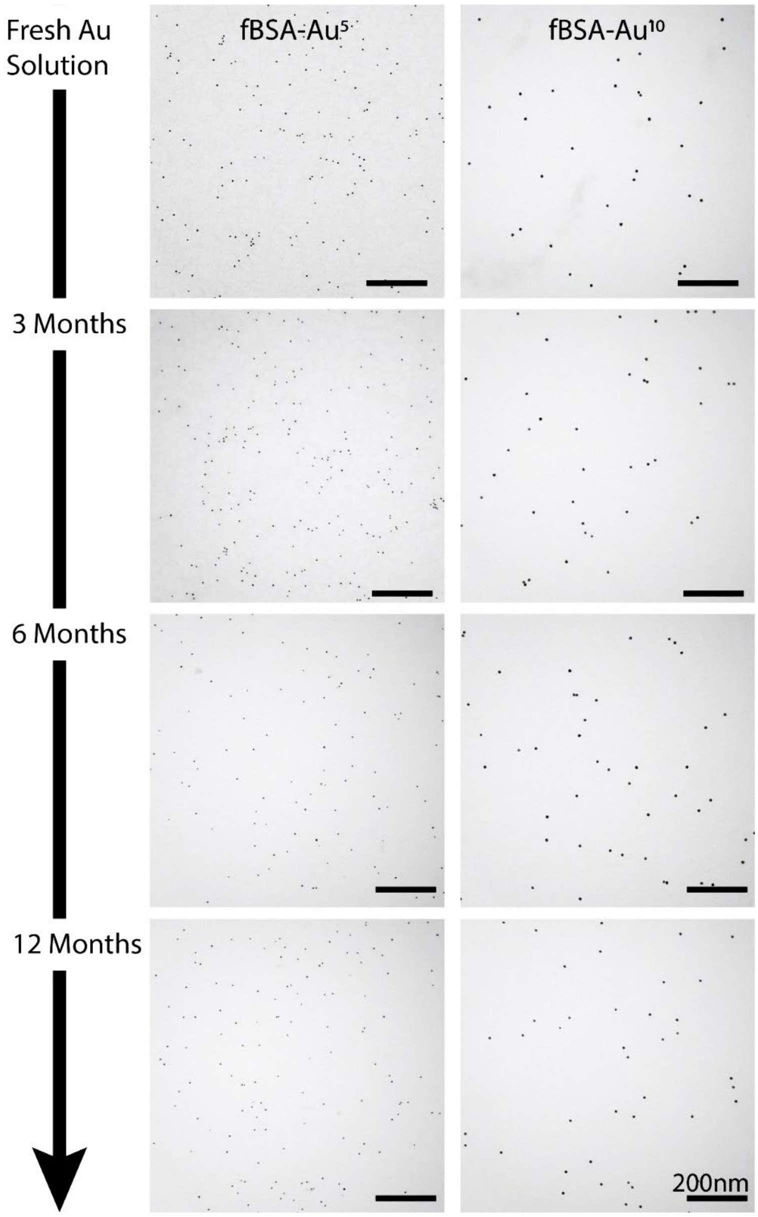

Supplementary Figure 1: fBSA-Au^{5} and fBSA-Au^{10} conjugates are stable in solution for 12 months. Related to Figure 1. TEM images of fBSA-Au^{5} (left column) and fBSA-Au^{10} (right column) particles deposited on TEM grids right after synthesis (Row 1), 3 months after synthesis (Row 2), 6 months after synthesis (Row 3), and 12 months after synthesis (Row 4). Note that even after 12 months there is no change in the size of the particles, and they remain dispersed with no clustering. *Scalebars: 200 nm*.

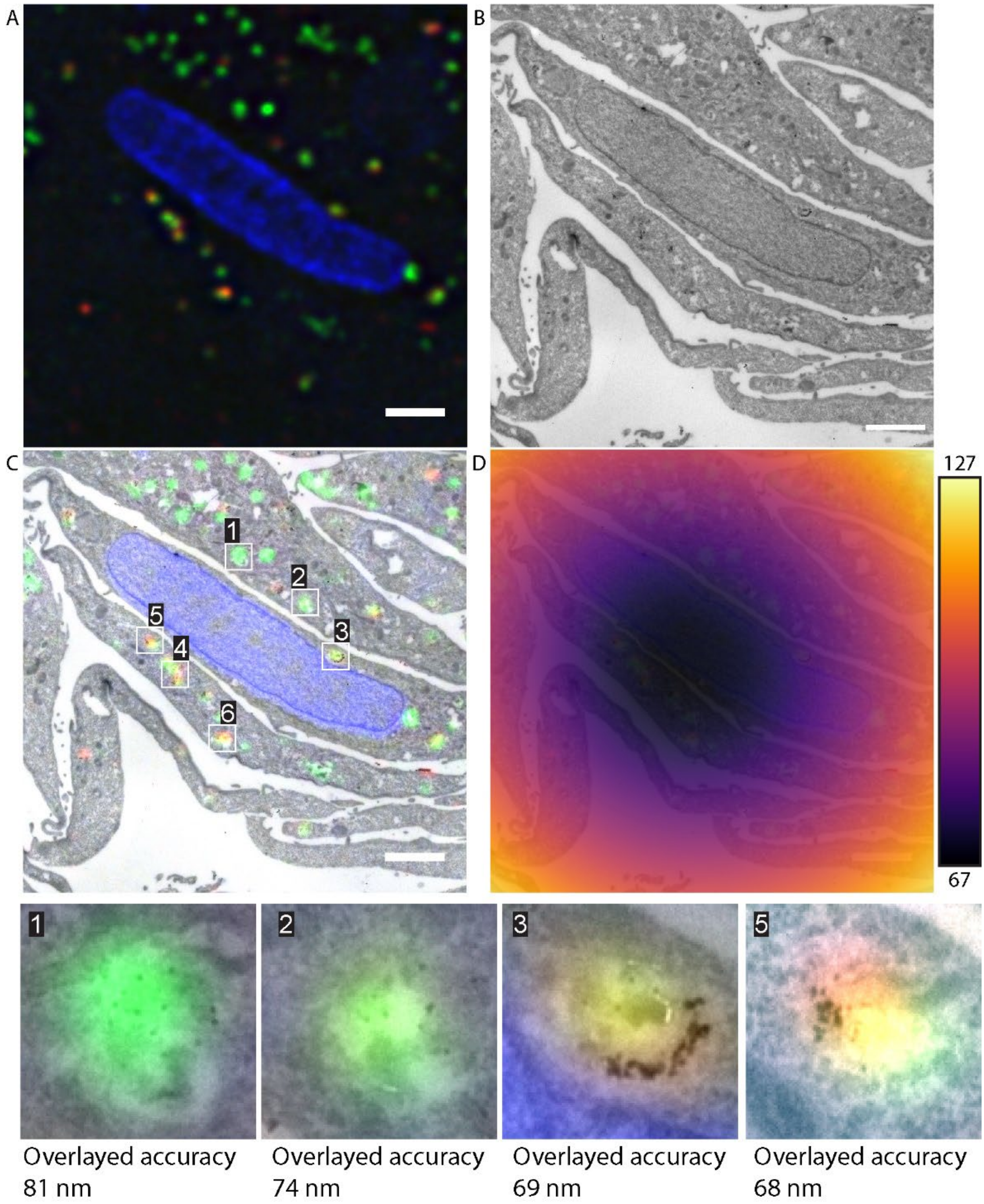

Supplementary Figure 2: fBSA-Au as fiducial enables high registration accuracy. Related to Figure 3. **(A)** FM data of region of interest on a ultrathin thawed cryosection prepared from cells with endocytosed fBSA-Au5 and immunolabelled for CD63 with Alexa488 and Au[10]. **(B)** EM region of interest. **(C)** Overlay of FM and EM data. **(D)** Quantification of registration error using ec-CLEM, showing regions of high accuracy (67 nm registration error) and lower accuracy (127 nm registration error). *Scalebars A, B, C, D: 2 µm.*

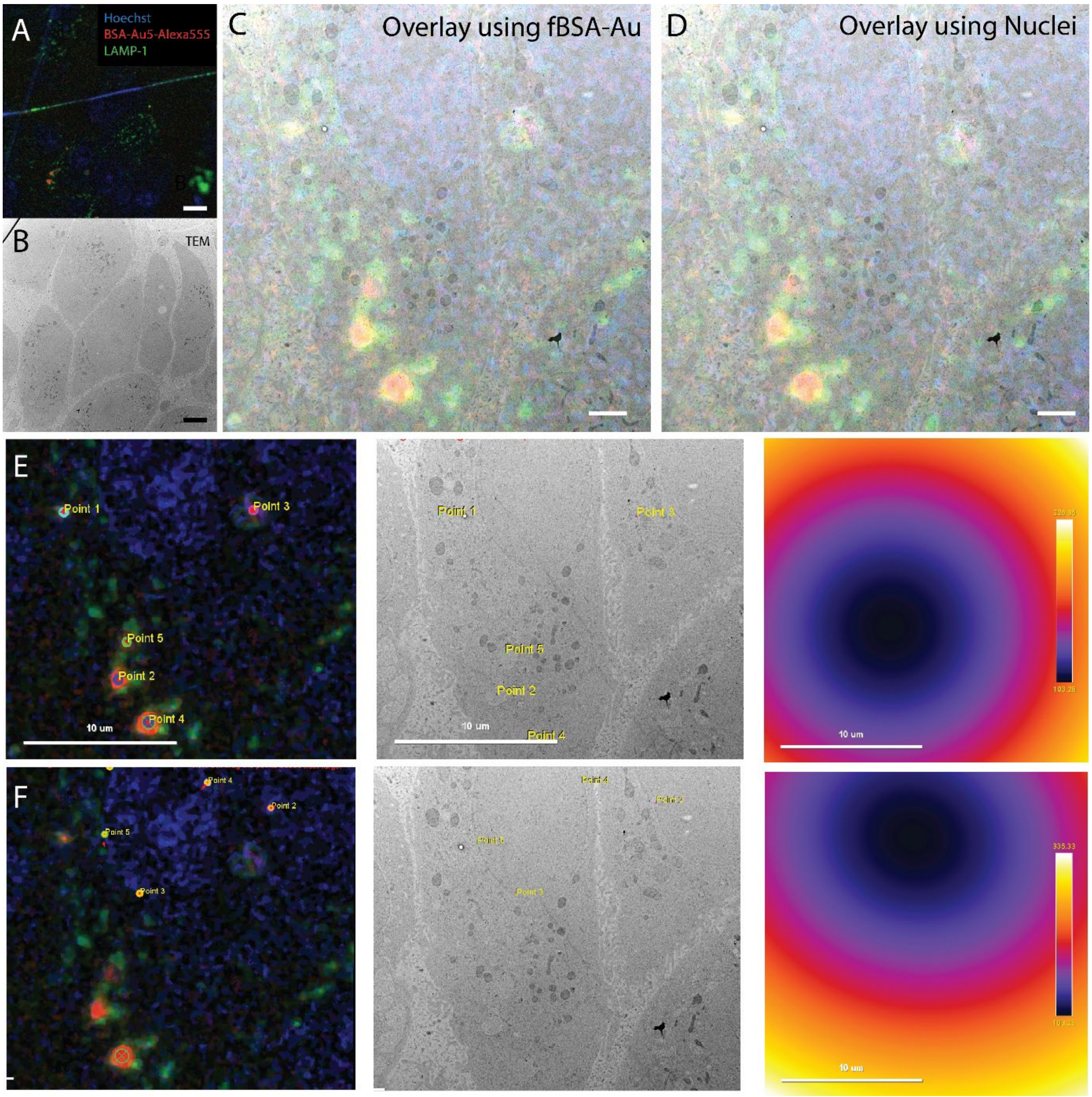

Supplementary Figure 3: Using fBSA-Au enables higher registration accuracy than using nuclei as a fiducial. Related to Figure3 and 4. (A) FM data of a region of interest on 100nm HM20 section prepared from cells with endocytosed fBSA-Au^5 and expressing LAMP-1-GFP. **(B)** EM image of the same region of interest. **(C)** Correlation of FM and EM data using five fBSA-Au^5 points in ec-CLEM software. **(D)** Correlation of FM and EM data using five nuclei edge points in and Hoechst signal in ec-CLEM software **(E)** Quantification of registration error in (C) using ec-CLEM, showing the accuracy ranges between 103 nm to 226nm. **(F)** Quantification of registration error in (D) using ec-CLEM, showing the accuracy ranges between 103 nm to 336nm. *Scalebars: (A)(B) 10 µm, (C)(D) 2 µm.*

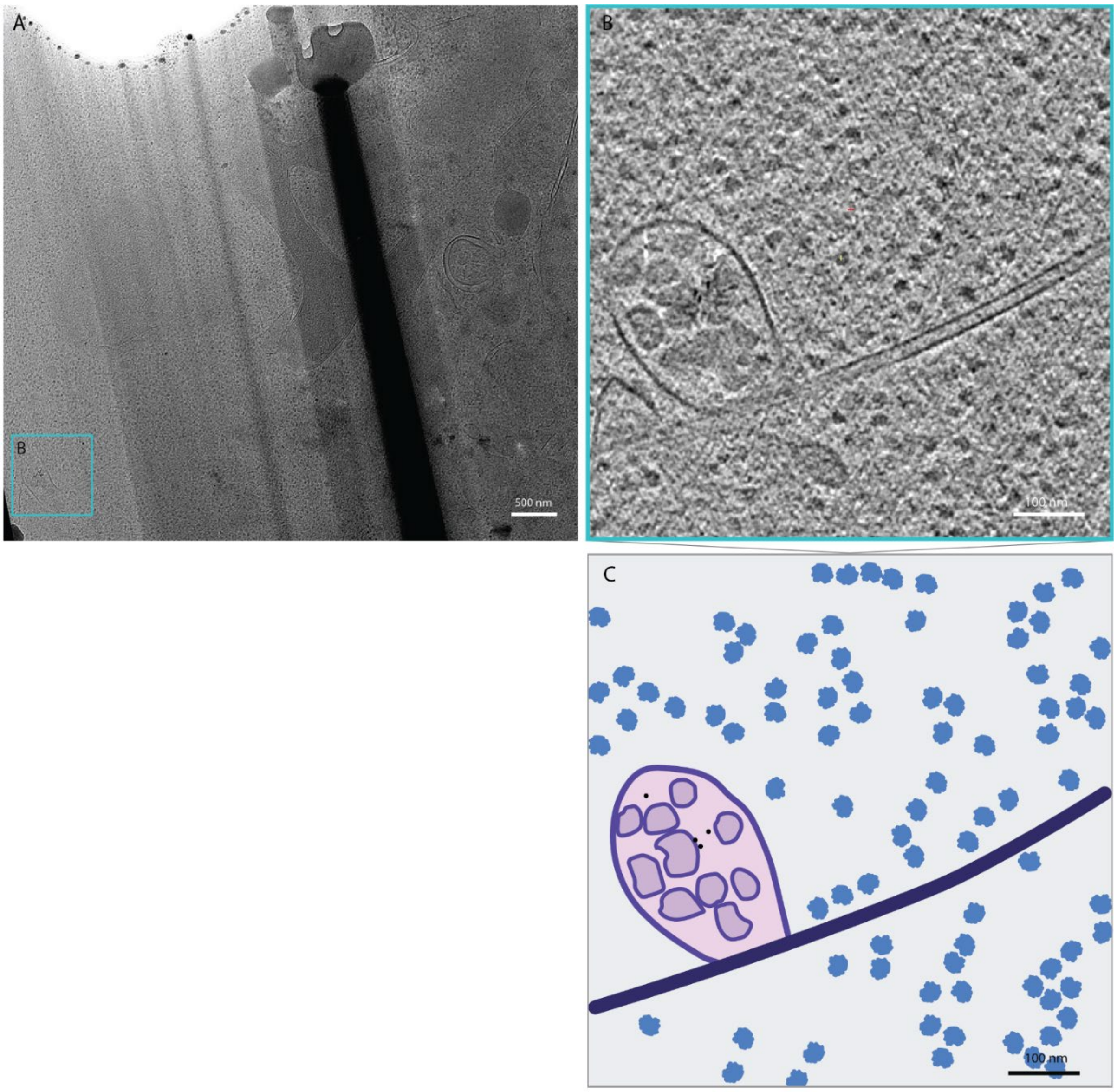

Supplementary Figure 4: Reconstruction of an endosomes containing fBSA-Au[5]. Related to Figure 7. **(A)** Overview cryo-TEM image of a lamella prepared by cryo-FIB-SEM. The region imaged with higher resolution is depicted with a blue square; and shown in (B). **(B)** An endosome bearing fBSA-Au[5] in its lumen. The contact site of the endosome with the microtubule cytoskeleton is visualized in cryo-ET. **(C)** Model for the endosome-microtubule interaction with lipid bilayer shown in purple, lumen in light pink, microtubule in dark blue, ribosomes in light blue and fBSA-Au[5] shown as black circles.*Scale bars: A: 500 nm; B,C: 100 nm.*
