# Supplementary material for: Bimodal endocytic probe for three-dimensional correlative light and electron microscopy
Source: Cell Rep Methods. 2022 May 16;2(5):100220. doi: 10.1016/j.crmeth.2022.100220 (PMC9142762; doi:10.1016/j.crmeth.2022.100220)
Supplement: Document S2. Article plus supplemental information [file mmc9.pdf]

Cell Reports Methods

Article

Bimodal endocytic probe for three-dimensional correlative light and electron microscopy

Graphical abstract

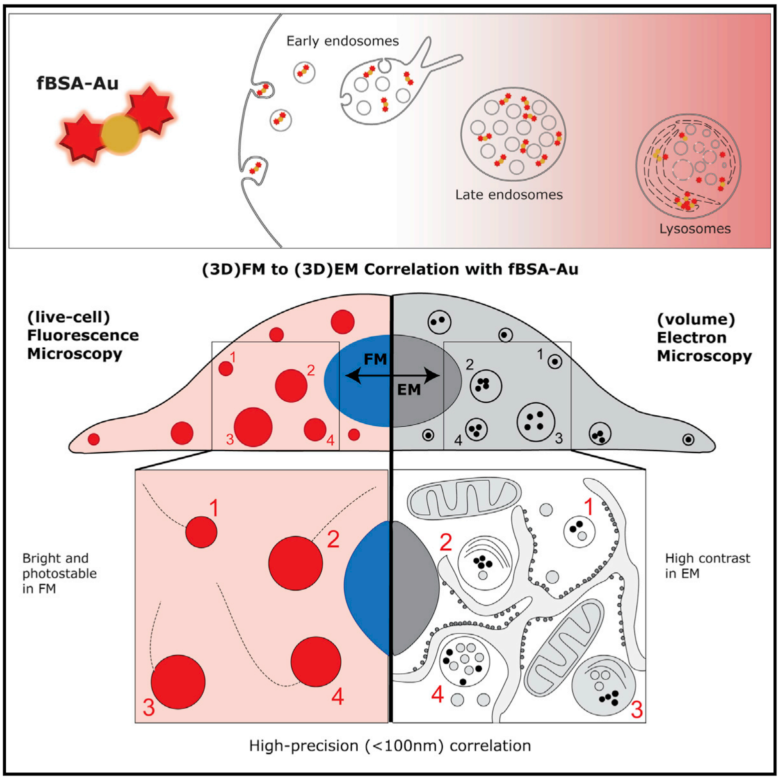

Authors

Job Fermie, Leanne de Jager, Helen E. Foster, ..., Hans C. Gerritsen, Judith Klumperman, Nalan Liv

Correspondence

n.liv@umcutrecht.nl

In brief

Fermie et al. present a fiducial marker, fBSA-Au, for high-precision correlation of 3D fluorescence microscopy (FM) and electron microscopy (EM) datasets. Endolysosomal uptake and trafficking of fBSA-Au generates a 3D pattern of intracellular landmarks for accurate FM-EM correlation (CLEM). fBSA-Au is demonstrated in a wide range of CLEM approaches.

Highlights

- fBSA-Au is a bimodal fiducial for precise registration of microscopy datasets
- Uptake of fBSA-Au into the endolysosomal system creates a 3D correlation map
- Suitable for key FM (live-cell, confocal, cryo) and EM (resin, immuno, cryo) methods
- High-accuracy correlation with fBSA-Au is shown in an array of (3D) CLEM approaches

Fermie et al., 2022, Cell Reports Methods 2, 100220
May 23, 2022 © 2022 The Author(s).
https://doi.org/10.1016/j.crmeth.2022.100220

Cell Reports Methods

CellPress
OPEN ACCESS

Article

Bimodal endocytic probe for three-dimensional correlative light and electron microscopy

Job Fermie,[1,2] Leanne de Jager,[3] Helen E. Foster,[4] Tineke Veenendaal,[1] Cecilia de Heus,[1] Suzanne van Dijk,[1] Corlinda ten Brink,[1] Viola Oorschot,[1,7] Lin Yang,[5] Wei Li,[5] Wally H. Müller,[6] Stuart Howes,[3] Andrew P. Carter,[4] Friedrich Förster,[3] George Posthuma,[1] Hans C. Gerritsen,[2] Judith Klumperman,[1] and Nalan Liv[1,8,*]

[1]Center for Molecular Medicine, University Medical Center Utrecht, Utrecht University, Utrecht, the Netherlands
[2]Molecular Biophysics, Debye Institute for Nanomaterials Science, Utrecht University, Utrecht, the Netherlands
[3]Structural Biochemistry, Bijvoet Centre for Biomolecular Research, Utrecht University, Utrecht, the Netherlands
[4]Medical Research Council Laboratory of Molecular Biology, Division of Structural Studies, Cambridge, UK
[5]Institute of Genetics & Developmental Biology, Chinese Academy of Sciences, Beijing, China
[6]Microbiology, Department of Biology, Utrecht University, Utrecht, the Netherlands
[7]Present address: Electron Microscopy Core Facility, EMBL Heidelberg, Heidelberg, Germany
[8]Lead contact
*Correspondence: n.liv@umcutrecht.nl
https://doi.org/10.1016/j.crmeth.2022.100220

MOTIVATION Correlative light and electron microscopy (CLEM) approaches are advancing our understanding of intracellular processes by linking molecular dynamics to 3D high-resolution structure. Quantitative application of most techniques, especially 3D CLEM, is currently hampered by limitations regarding accurate registration of fluorescent signals to electron microscopy (EM). Directly addressing this limitation, we describe fluorescent BSA-gold (fBSA-Au), a bimodal endocytic tracer, as a fiducial marker for 2D and 3D CLEM applications. The fiducial exploits a cell's own endolysosomal system to create a well-defined 3D pattern of landmarks and provides a high-precision (<100 nm) registration between fluorescence microscopy (FM) and EM datasets.

SUMMARY

We present a bimodal endocytic tracer, fluorescent BSA-gold (fBSA-Au), as a fiducial marker for 2D and 3D correlative light and electron microscopy (CLEM) applications. fBSA-Au consists of colloidal gold (Au) particles stabilized with fluorescent BSA. The conjugate is efficiently endocytosed and distributed throughout the 3D endolysosomal network of cells and has an excellent visibility in both fluorescence microscopy (FM) and electron microscopy (EM). We demonstrate that fBSA-Au facilitates rapid registration in several 2D and 3D CLEM applications using Tokuyasu cryosections, resin-embedded material, and cryoelectron microscopy (cryo-EM). Endocytosed fBSA-Au benefits from a homogeneous 3D distribution throughout the endosomal system within the cell, does not obscure any cellular ultrastructure, and enables accurate (50–150 nm) correlation of fluorescence to EM data. The broad applicability and visibility in both modalities makes fBSA-Au an excellent endocytic fiducial marker for 2D and 3D (cryo)CLEM applications.

INTRODUCTION

The ability to visualize the spatial and temporal characteristics of organelles and proteins is crucial in many areas of cell biology. Fluorescence microscopy (FM) is highly sensitive, has a large toolbox to simultaneously analyze multiple cellular parameters, and can be used to examine the dynamics of processes in live cells. A limitation, however, is its inability to report the structural context underlying the molecular localization patterns. For this information, electron microscopy (EM) is the method of choice. Its greater resolution uniquely allows us to locate proteins in the cellular context because of its ability to directly visualize membranes and other macromolecular structures. However, specific proteins or structures of interest cannot always be discerned by morphology alone, and additional, electron-dense labeling methods are required to visualize them. Various labeling methods are available for EM, such as immunolabeling with colloidal gold (Horisberger and Rosset, 1977; Slot and Geuze, 2007) or peroxidase-based methods generating osmiophilic precipitates (Deerinck et al., 1994; Gaietta et al., 2011; Martell et al., 2012). These strategies do have limitations because the electron-dense precipitates generated by peroxidase reactions can obscure ultrastructural details, and immunolabels have inherently limited penetration into specimens (Stierhof et al., 1986;

This is an open access article under the CC BY license (http://creativecommons.org/licenses/by/4.0/).

Stierhof and Schwarz, 1989). Correlative light and EM (CLEM), which integrates the data from FM and EM on a single sample, overcomes these limitations. CLEM uses the large reporter diversity and sensitivity from FM to provide protein localization information and register this information to high-resolution morphological data without the limitations of EM labeling.

One of the main challenges of CLEM is to accurately assign the fluorescent label from FM to the corresponding feature in EM. Retracing specific fluorescent cells or subcellular structures in a large dataset requires reference points that must be easily identifiable in both modalities. These can be naturally formed landmarks, such as branching blood vessels and unique cell shapes (Karreman et al., 2016), artificial marks on the sample support (Polishchuk et al., 2000; Müller-Reichert et al., 2007; van Rijnsoever et al., 2008; Verkade, 2008), or the sample itself (Vicidomini et al., 2008; Bishop et al., 2011; Bushby et al., 2012; Karreman et al., 2016). These landmarks are simply too big and do not provide the required registration accuracy to correlate fluorescence signals to individual subcellular structures with nanometer precision. In addition, they may not always be present in quantities sufficient for accurate registration. For these applications, correlation is ideally achieved through artificial fiducials (Roth et al., 1980; Powell et al., 1998; Kukulski et al., 2011; Watanabe et al., 2011; Kopek et al., 2012; Takizawa et al., 2015; Fokkema et al., 2018). Fiducials are particles easily visible in FM and EM that are small enough to not obscure morphological details (<100 nm). A variety of particles have been developed and used in correlative methods, achieving correlation accuracies well below 100 nm (Giepmans et al., 2005; Kukulski et al., 2011; Watanabe et al., 2011; Kopek et al., 2012). These approaches work most efficiently in 2D CLEM applications, where fiducials are commonly applied to the surface of a substrate; i.e., on a coverslip or the formvar layer of an EM grid. However, with the increasing popularity of live-cell and 3D CLEM applications (Hoffman et al., 2020), there is a pressing need for strategies to distribute fiducials intracellularly in 3D, which is still unaddressed.

To guarantee accurate registration in 3D CLEM applications, fiducials should be present throughout the entire volume of interest. The endosomal system naturally provides a 3D network of vesicular structures throughout the cell and is easily reached and manipulated from the extracellular environment. This property prompted us to explore the endolysosomal system as a means to distribute fiducials throughout the cell. Endocytic tracers such as dextran, albumins, or nanoparticles conjugated to fluorophores or colloidal gold are commonly used to mark endosomal compartments. One of the most applied endocytic probes, bovine serum albumin (BSA), efficiently labels early endosomes, late endosomes, and (auto)lysosomes and can be conjugated to a variety of fluorophores and colloidal particles (Geoffroy and Becker, 1984; Hanaki et al., 2003; Zhang and Hensel, 2013). BSA conjugates to fluorophores or gold particles have been used in the past for FM or EM approaches. These conjugates are highly visible and have no cytotoxicity, even after prolonged chase times (Connor et al., 2005; Chithrani et al., 2006; Zhang and Hensel, 2013). We reasoned that fluorescently labeled BSA conjugated to electron-dense particles could function as an efficient fiducial for 3D CLEM, but no such probe is available to date.

Here we present an endocytic tracer consisting of 5- or 10-nm colloidal gold stabilized with fluorescently labeled BSA (fBSA-Au^5 and fBSA-Au^{10}, respectively). We demonstrate applicability of fBSA-Au as a fiducial marker in a variety of 3D CLEM approaches using Tokuyasu on-section CLEM, resin embedding 3DCLEM, and targeted lamella preparation for cryoelectron microscopy (cryo-EM). We show that the small size of the conjugate enables efficient endocytosis, whereas the high atomic number of the gold colloids ensures good visibility in EM. Importantly, the bimodal nature of the tracer guarantees accurate registration of FM and EM data, by which endocytic compartments containing multiple fluorescent gold colloids serve as fiducial landmarks. Finally, the uniform size of the gold particles ensures excellent compatibility with immuno-EM (double) labeling strategies (Slot and Geuze, 1981). These benefits make fBSA-Au a highly useful tool for 2D and 3D (cryo) CLEM applications.

RESULTS

fBSA-Au particles are stable and monodisperse

As an electron-dense core of the bimodal probe, we chose colloidal gold particles nominally sized at 5 or 10 nm because of their small size and high visibility in EM. The small size guarantees efficient endocytosis, precise correlations, and compatibility with other immunoEM methods. Using the protocol developed previously (Slot and Geuze, 1981, 1985), we synthesized monodisperse colloidal particles of different sizes (Figures 1A, 1D, and 1G). The resulting gold colloids were stabilized with BSA-Alexa 555 and purified using centrifugation on a glycerol gradient to remove unbound BSA-Alexa 555. This process yielded BSA-conjugated particles of uniform size, with fBSA-Au^5 averaging 5.7 ± 0.7 nm (n = 220) and fBSA-Au^{10} averaging 9.1 ± 0.5 nm (n = 180) in diameter, as measured using transmission EM (TEM) (Figures 1A, 1B, 1D, and 1E). BSA binding to the particles could not be detected using TEM, likely because of the low electron contrast of the BSA, which is poorly visible in EM without heavy metal contrasting (Figure 1H).

Stabilization of the gold particles with BSA-Alexa 555 resulted in a detectable shift in size distribution, indicating binding of BSA-Alexa 555 to the gold particles without forming aggregates of larger sizes. To test clustering behavior, size distributions of the particles were recorded using dynamic light scattering (DLS) (Figures 1C and 1F). We found that the "bare" and "functionalized" particles remain non-clustered in solution. The schematic in Figure 1G shows the relative sizes of a 5-nm Au particle, BSA molecules (7.1–7.5 nm), and Alexa 555 molecules with respect to each other (Waterhouse et al., 2018). We found that fBSA-Au conjugates remain stable in solution over extended periods of time, showing no signs of clustering or precipitation after several (>12) months of storage at 4°C (Figure S1). These data show that colloid gold particles can be functionalized with BSA-Alexa 555, after which the resulting conjugates remain monodisperse, uniformly sized, and stable in solution.

fBSA-Au is efficiently endocytosed and transported by the endosomal system

After characterization of the size and clustering behavior of the conjugates, we assessed the feasibility of fBSA-Au for cellular

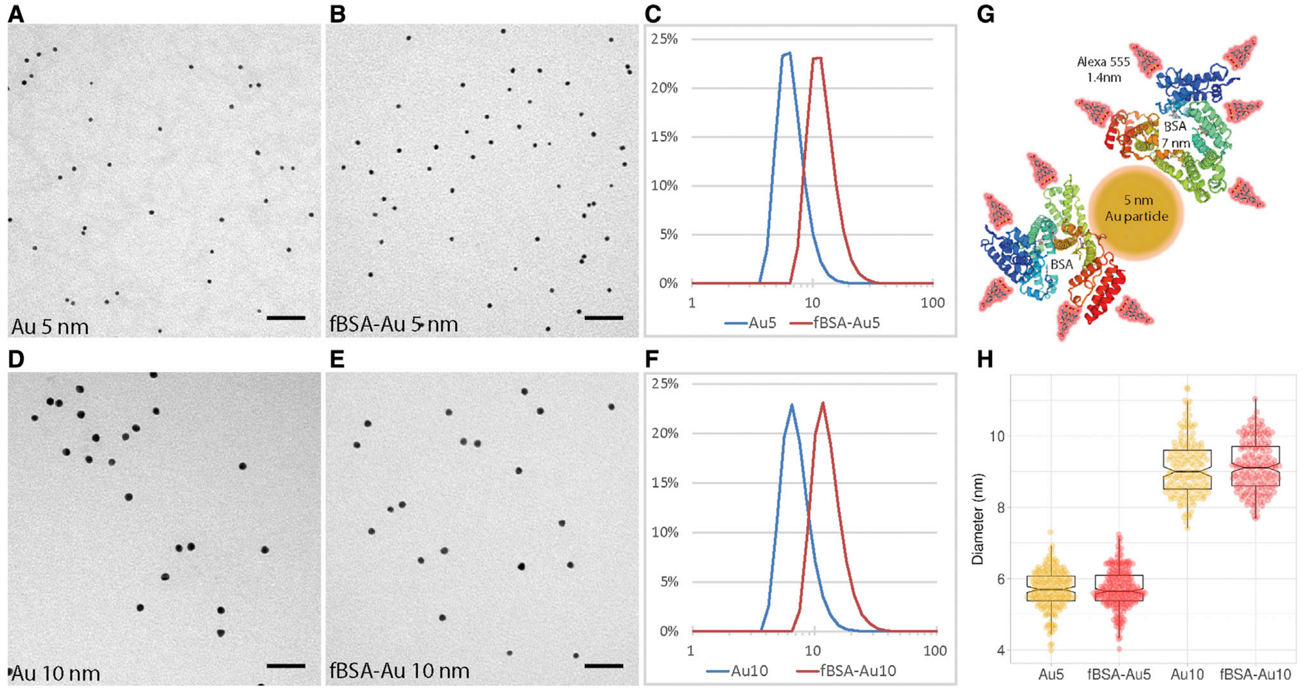

Figure 1. Characterization of synthesized fBSA-Au conjugates

(A and B) Representative TEM micrographs of 5-nm colloidal gold, showing the monodisperse nature of the particles before (A) and after (B) BSA functionalization.

(C) DLS measurements showing the size distribution of 5-nm colloidal gold and BSA-Alexa^{555} functionalized particles. Functionalization causes a shift in size distribution but does not induce larger aggregates.

(D and E) Representative TEM micrographs of 10-nm colloidal gold before (D) and after (E) BSA functionalization.

(F) DLS measurements showing the size distribution of 10-nm colloidal gold and BSA-Alexa^{555} functionalized particles. BSA functionalization causes a shifted size distribution but does not induce larger aggregates.

(G) Representative schematic showing the relative sizes of BSA (7.1 nm), Alexa 555 (1.4 nm), and Au particles (5 nm).

(H) Sizing of Au^5 and Au^{10} particles determined by TEM. Au^5 and Au^{10} are homogeneously sized. Unlike in DLS measurements, the apparent size of the functionalized particles does not increase after BSA functionalization, likely because of the poor visibility of the electron-lucent BSA.

Graphs depict mean ± SD Scale bars, 50 nm.

CLEM experiments. To be useful as an endocytic CLEM probe, the probes should be non-toxic to cells, efficiently endocytosed, and brightly fluorescent throughout the experiment.

We first examined the uptake efficiency and localization of internalized fBSA-Au^5 using FM. After endocytosis, BSA conjugates are predominantly targeted to the degradative path of the endosomal system, where they accumulate in late endosomes and lysosomes (Pols et al., 2013; Jonker et al., 2018). We incubated HeLa cells with fBSA-Au^5 for 3 h to label the entire endosomal pathway, including lysosomes, the terminal compartments of the endocytic route (Pols et al., 2013; Zhang and Hensel, 2013). After uptake, cells were chemically fixed and immunolabeled for EEA1 and LAMP-1 to mark early endosomal and late endosomal/lysosomal compartments, respectively (Figure 2A). The cells were imaged using FM, and colocalization between channels was analyzed using a spot detection-based colocalization plugin in Fiji (see STAR Methods for details). This showed that internalized fBSA-Au^5 results in the appearance of strongly fluorescent spots that colocalize with EEA1 and LAMP-1 (Figure 2B). Line profile (Figure 2C) and colocalization analysis (Figure 2D) show that the majority of fBSA-Au^5 colocalizes with EEA1 and LAMP-1, and only a negligible part (3.8%) does not colocalizing with these early or late endolysosomal markers. The data show that fBSA-Au^5 is efficiently taken up by cells and transported through the endolysosomal pathway, reaching early and late endocytic compartments.

To examine whether fBSA-Au is compatible with application of commonly used endocytic markers, we incubated HeLa cells with dextran 488 and fBSA-Au^{10} for 3 h (Makarow, 1985). After incubation, cells were fixed and examined by FM. We performed uptake experiments with 5- and 10-nm Au particles and observed no difference in their endocytosis and trafficking. We found that fBSA-Au^{10}, like fBSA-Au^5, is readily taken up, resulting in the appearance of brightly fluorescent spots (Figures 2E and 2F). Line profiling shows that the fluorescent signals of dextran and fBSA-Au^{10} largely colocalized; on average, 80% of dextran 488-positive spots also contained fBSA-Au^{10} (Figure 2G). This indicates that the fBSA-Au probe can be combined with generic endocytic markers and shows that both probes follow the same endocytic route to lysosomes.

Finally, we examined whether the fBSA-Au probes can be applied to live cell imaging, which requires bright and stable probe fluorescence. After incubating cells for 3 h with fBSA-Au^5, coverslips were imaged live with wide-field FM. This

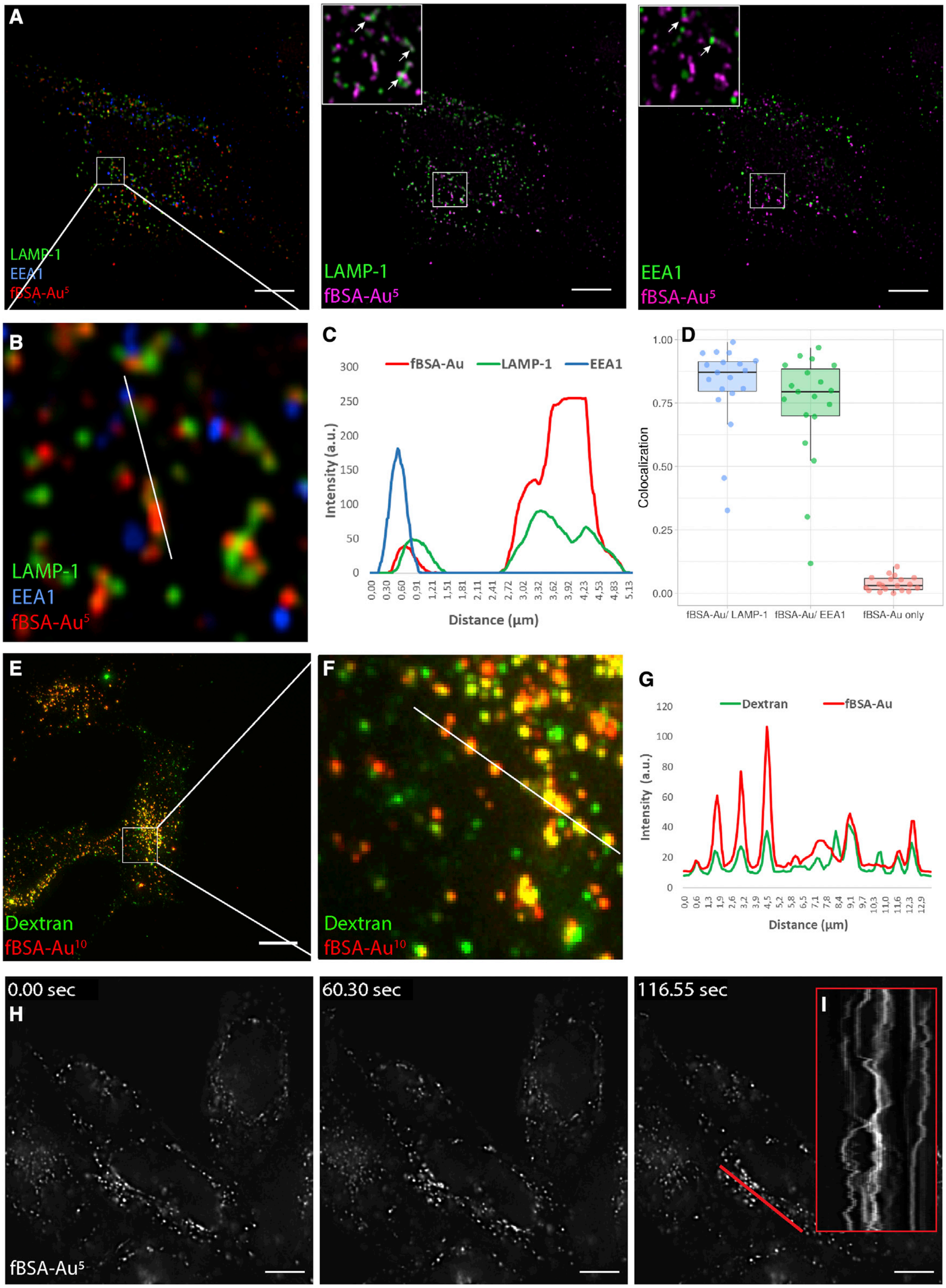

Figure 2. fBSA-Au is a highly fluorescent, efficiently internalized endocytic tracer

(A) Fluorescence images of fixed HeLa cells incubated with fBSA-Au^5 and immunolabeled for EEA1 and LAMP-1. fBSA-Au^5 is visible in EEA1- and LAMP-1-positive compartments; i.e., throughout the endolysosomal system.

(B–D) Magnification of the white square in (A) (B), depicted line profile (C), and colocalization analysis (D) show that internalized fBSA-Au^5 colocalizes with EEA1 and LAMP-1.

(legend continued on next page)

showed that fBSA-Au^5-positive fluorescent spots actively moved in the cells (Figure 2I, kymograph along the red line) and remained brightly fluorescent over 250 acquired frames during live-cell imaging (Figure 2H; Video S1). We conclude that fBSA-Au is an appropriate probe for FM studies, including live-cell imaging. It is efficiently taken up by cells and distributed throughout the endosomal pathway.

fBSA-Au is an efficient fiducial for 2D on-section CLEM

After validating endocytosis of fBSA-Au^5 and fBSA-Au^{10}, we tested the applicability of these conjugates for CLEM experiments. First, we tested a 2D on-section CLEM setup using ultrathin Tokuyasu cryosections, the most sensitive method for immuno-EM (van Rijnsoever et al., 2008; Vicidomini et al., 2008, 2010; Mari et al., 2014). Because cryosections, in contrast to resin sections, show no fluorescent background signal, and epitopes are generally well preserved in this approach, they are uniquely suitable for imaging by FM and EM. CLEM applications using cryosections often involve on-section labeling with a fluorescently tagged antibody that is also marked by colloidal gold (Geuze et al., 1981; Oorschot et al., 2014). The fluorescence signal obtained from ultrathin cryosections is limited by the thickness of the sections, which is approximately 70 nm. Therefore, fBSA-Au^5 fluorescence must be sufficiently intense to be used as fiducial in this approach.

HeLa cells were incubated with fBSA-Au^5 for 3 h, and then cells were prepared for cryosectioning and immunolabeling according to our well-established protocol (Geuze et al., 1981; Slot and Geuze, 2007). We chose to use fBSA-Au^5 for these and the following experiments because its small size enables easy distinction from commonly used 10- or 15-nm-sized immunogold labels. Ultrathin cryosections prepared from these HeLa cells were immunolabeled with a primary monoclonal antibody against CD63, a marker for late endosomes and lysosomes, followed by secondary Alexa 488-tagged antibody and 10-nm-sized protein A-gold. By FM, we found that the Alexa 555 fluorophore of fBSA-Au^5 withstood the preparation steps of cryosections, was preserved, and provided a detectable fluorescent signal in the ultrathin cryosections after aldehyde fixation, cryoprotection, and plunge freezing. The fBSA-Au^5 signal was clearly visible in 70-nm thin sections, where it frequently colocalized with CD63 labeling (Figure 3A). We then selected regions of interest (ROIs) for imaging in TEM and registered fBSA-Au^5 fluorescent spots to the EM ultrastructure (Figures 3B and 3C). We performed an initial correlation based on recognizable features, such as nuclei and cell shapes (Figures 3A and 3B), to find back our cells of interest. Then we used the fBSA-Au^5 fiducials to zoom in and correlate subcellular structures in FM and EM. We found that Alexa 555-labeled compartments in FM always contained 5-nm gold (Figure 3D, arrowheads) upon EM inspection. The reverse was also valid; gold-containing endosomes and lysosomes identified in EM always contained Alexa 555 fluorescence when correlated back to the FM image (Figure 3D). This showed that fBSA-Au^5 fluorescence precisely correlates with endosomal compartments containing Au^5-positive compartments visible by EM.

The intensity of the fBSA-Au^5 fluorescent spots correlated well with the number of gold particles in a compartment; bright fluorescent spots correlated with compartments with large numbers of gold particles, whereas small but readily detectable fluorescent spots correlated with individual particles or small clusters of gold (Figure 3D). This fluorophore-gold correlation is possible because the fluorescent and gold signals are from the same section, containing precisely the same number of fBSA-Au^5 particles. To establish the resolution of the correlation approach, we selected the center of fBSA-Au^5 fluorescence for a given organelle on FM images and the center of the same organelle in EM images (Paul-Gilloteaux et al., 2017). By doing this, we reached suborganelle accuracy registration. For example, manual picking of 15 pairs of FM and EM fBSA-Au^5 signals followed by semi-automated correlation with a previously established correlation algorithm, ec-CLEM (Paul-Gilloteaux et al., 2017; Figure S1), resulted in a registration accuracy between 60 and 130 nm. This high accuracy of correlation also enabled us to correlate organelles labeled for CD63 (arrows) but lacking fBSA-Au^5 (Figure 3D, panel 1). We also compared the correlation accuracy of FM and EM data of the GFP-LAMP-1 signal in high-pressure-frozen and HM20-embedded HeLa cells (Figure S3) in ec-CLEM software, using fBSA-Au^5 or the Hoechst signal and the edges of nuclei. We used 5 points each for registration with the fBSA-Au and nucleus signal. Quantification of registration error and comparing the accuracy of this limited set of points using ec-CLEM showed that accuracy ranged between 103 and 226 nm for fBSA-Au particles and between 103 and 337 nm using the nuclear signal.

These data show that the fBSA-Au^5 probe correlates with 100% efficiency between the FM and EM modalities and, by distribution and intensity, can be reliably used as a fiducial marker to overlay FM onto EM images. Mapping the constellation of multiple fBSA-Au-labeled compartments provides the resolution to correlate fluorescently labeled structures that lack fBSA-Au, which allows broad application of the probe to endolysosomal as well as other structures.

fBSA-Au is a suitable fiducial for 3D correlative FM-electron tomography

To extend application of fBSA-Au to 3D on-section CLEM, we next made semi-thin (~350-nm) cryosections and prepared these for FM and electron tomography (ET). ET is a unique tool to view 3D nanometer-scale details in the cellular context (Kremer et al., 1996). ET of semi-thin cryosections is a powerful approach for high-resolution 3D ultrastructural

(E) Fluorescence images of fixed HeLa cells incubated with fBSA-Au^{10} and dextran-Alexa 488.
(F and G) Magnification (F) and intensity profile (G) over the depicted lane show that fBSA-Au^{10} is readily endocytosed and largely colocalizes with dextran.
(H) Stills from Video S1 of live HeLa cells loaded with fBSA-Au^5. fBSA-Au^5 has sufficient fluorescence intensity to employ time-lapse experiments with high temporal resolution.
(I) Kymograph over the indicated red line shows the x-t scan along the depicted red line.
Scale bars, 10 μm.

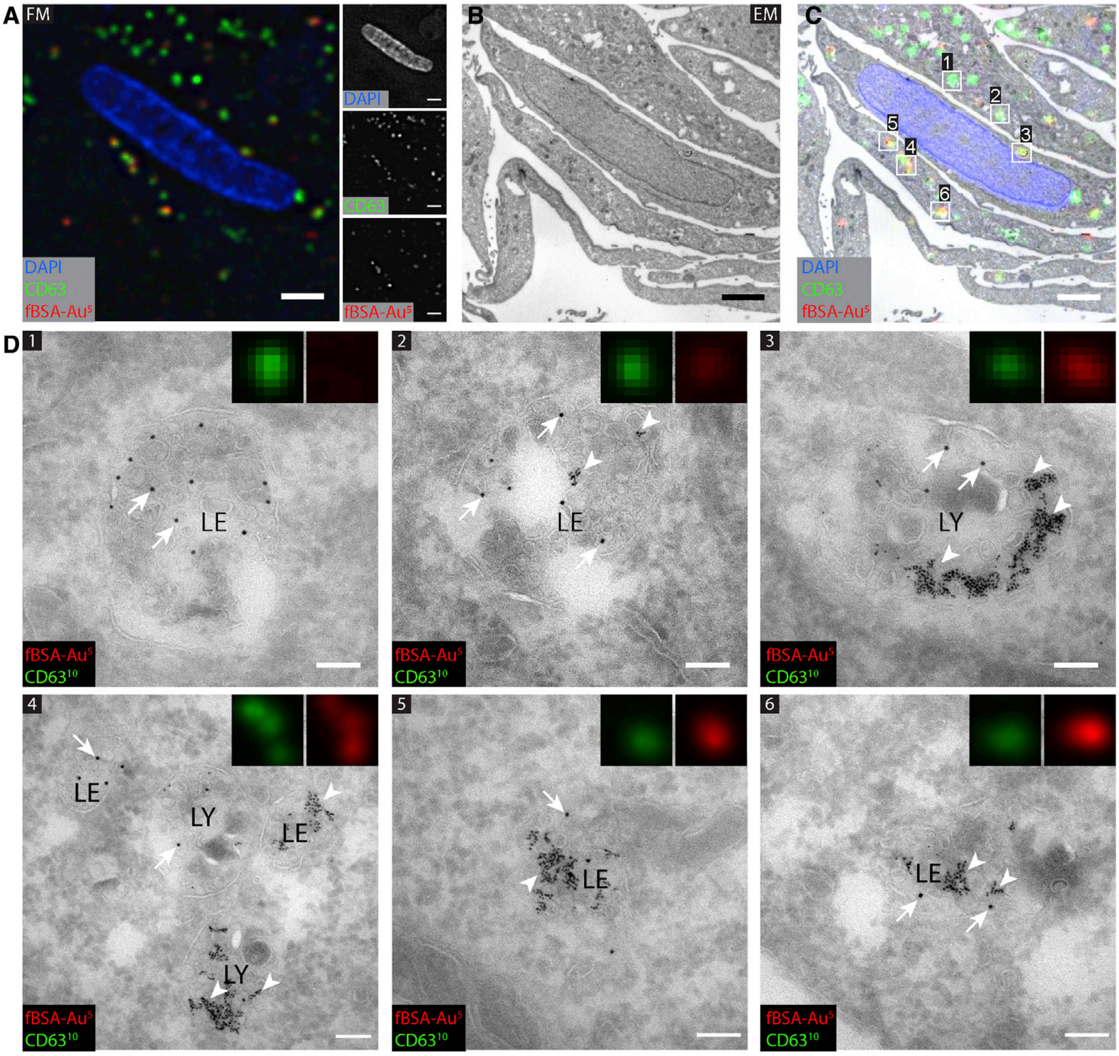

Figure 3. CLEM of CD63-positive, fBSA-Au-containing compartments in HeLa cells

(A) FM image of an ultrathin cryosection. Shown is the region of interest (ROI) with CD63 immunolabeling (Alexa 488 and Au^{10}) and Alexa 555 fluorescence of internalized fBSA-Au^5.

(B and C) EM of the ROI (B) and overlay of FM and EM images (C), showing high registration accuracy between modalities.

(D) High-magnification EM of fBSA-Au^5 (arrowheads) containing organelles labeled for CD63 (10-nm gold, arrows). All selected compartments are positive for CD63 (10-nm gold, arrows). Insets: magnification of CD63 and fBSA-Au^5 fluorescence of the selected compartments; width of insets, 800 nm. The intensity of fBSA-Au^5 Alexa 555 fluorescence corresponds to the number of gold particles per compartment. (1) and (2) show late endosomes (LEs) containing many intraluminal vesicles and no (1) or little (2) fBSA-Au^5 label. (3) shows a lysosome (LY) with clusters of fBSA-Au^5 gold particles. (4) shows several LEs and LYs containing varying levels of fBSA-Au^5. (5) and (6) show LEs heavily loaded with fBSA-Au^5 correlating with intense red fluorescence.

Scale bars, 2 μm (A–C) and 100 nm (D).

analysis of organelles (Zeuschner et al., 2006; Franke et al., 2019).

HeLa cells that were incubated for 3 h with fBSA-Au^5 were prepared as stated in the previous section, and then ~350-nm cryosections were prepared. The sections were thawed and immunolabeled for the lysosomal marker LAMP-1, followed by secondary labeling with Alexa 488-tagged antibodies and 10-nm-sized protein A-gold. In contrast to fBSA-Au^5 added to whole cells for internalization, antibody-based LAMP-1 labeling is applied to sections. The antibodies can penetrate cryosections, as opposed to resin-embedded materials, but gold particles mostly remain at the section surface (Stierhof et al., 1986; Stierhof and Schwarz, 1989). Hence, there is no direct correlation between the intensities of FM and EM labeling of LAMP-1(Van E. et al., 2017).

By FM, fBSA-Au^5 was readily detected as distinct spots that partially colocalized with LAMP-1 (Figure 4A). The overall fluorescence intensity of fBSA-Au^5 was higher than in 70-nm cryosections (Figures 3 and 4), which corresponds to a higher number of fBSA-Au^5 particles in the increased Z volume of 350 nm. We then selected regions with fBSA-Au^5 fluorescence for correlation with ET. ET image acquisition involves tilting of a sample

CellPress
OPEN ACCESS

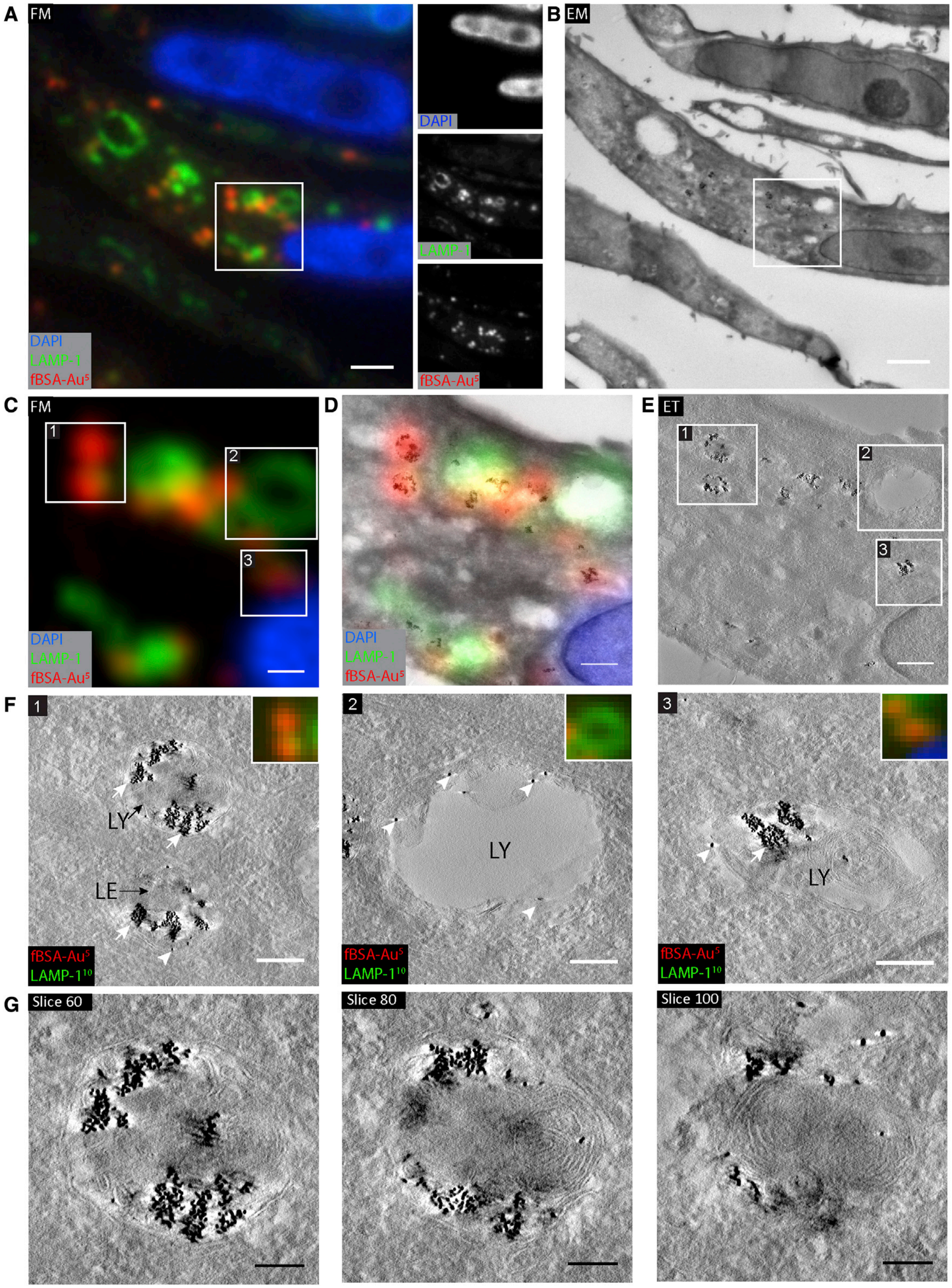

Figure 4. 3D CLEM of 350 nm cryosections using ET

HeLa cells were incubated with fBSA-Au^5 fiducials (3 h) and immunogold labeled for LAMP-1 (10-nm gold).

(A) FM image of a 350-nm-thick cryosection with the selected ROI for ET highlighted by the white box. Insets show separate channels for the used fluorophores.

(B) EM of the same region shown in (A). At this magnification, clusters of endocytosed fBSA-Au^5 gold particles are visible, allowing rapid correlation from FM to EM. The ROI selected for ET is shown in the white box.

(legend continued on next page)

around 1 axis or 2 axes, and then a 3D reconstruction is generated based on back-projection algorithms (Kremer et al., 1996). Because ET is generally only performed on a small ROI, it is of particular importance to select the proper ROI for image acquisition. In semi-thin sections, the increased thickness causes more scattering of electrons and obscures visibility of structures by 2D TEM, which hampers selection of ROIs. The electron-dense gold particles of fBSA-Au^5 were very well visible by 2D TEM, which greatly facilitating selection of an ROI. After identifying the proper ROIs, tilt images were collected by ET (Figures 4B–4D), in which individual organelles were registered from FM to 3D EM. The correlation accuracy of this approach ranged between 60 and 200 nm, as determined using ec-CLEM, which is at the same level as for 2D CLEM. These data show that, in semi-thin sections, the fBSA-Au^5 probe can be used at the mesoscale—to find back cells and ROIs—and at the nanometer level—to correlate individual organelles.

After tomogram reconstruction, we examined the morphology of the correlated structures. ROI 1 (Figures 4C–4F) showed a collection of fBSA-Au^5 particles present in a late endosome and a lysosome. Multiple virtual slices through the lysosome showed that fBSA-Au^5 is evenly distributed throughout the organelle (Figure 4G). ROI 2 contained a large, LAMP-1-positive organelle negative for fBSA-Au^5. The corresponding ET image shows only sparse LAMP-1 gold-labeling on the section surface. Thus, despite the absence of fBSA-Au^5 and sparse immunogold labeling, these organelles could be marked as LAMP-1 positive, as detected by FM labeling registered to EM. These data show that the intensity of the FM signal and the accuracy of the correlation procedure overcomes the need to label fluorescently tagged antibodies with an additional gold tag (van der Beek et al., 2022). ROI 3 showed a typical lysosome containing ample fBSA-Au^5 particles. The 5-nm gold particles are visible throughout the volume of the tomograms (Figures 4F and 4G) because of internalization of fBSA-Au^5 prior to fixation. The intracellular 3D distribution of fBSA-Au^5 allowed correlation of structures throughout the section (Figure 4G), which is an important improvement over fiducials that reside on the surface of sections.

fBSA-Au as an endocytic fiducial for correlative live-FM and ET of resin-embedded cells

Many CLEM approaches use resin embedding for EM. In these applications, the flow of experiments generally involves recording of fluorescence signals in live or fixed 3D samples (i.e., not sections as above), after which the sample is infiltrated/stained with heavy metals for increased EM contrast and embedded in epoxy or acrylic resin. Then (serial) ultrathin or semi-thin sections are made, which are collected for (serial) TEM or ET and correlated with the FM data. This type of correlative approach is sensitive to distortions between FM and EM datasets because staining, dehydration, and embedding steps are performed after FM imaging. 3D CLEM approaches (serial sectioning and/or ET) introduce an additional level of challenge in the z dimension because of the disparity in axial resolution between FM and EM. We reasoned that fBSA-Au fiducials, because of their unique 3D distribution, would significantly ease such 3D CLEM correlations and tested this by making ETs of serial semi-thick sections.

HeLa cells grown on patterned glass coverslips were incubated for 3 h with fBSA-Au^5, fixed, and imaged using FM. Cells were recorded relative to the pattern on the coverslips to facilitate successive X-Y correlation to EM (Polishchuk et al., 2000), as commonly used in volume-CLEM applications (Russell et al., 2016; Fermie et al., 2018). The volume-CLEM approach is outlined in Figure 5A. A fluorescent z stack of the cell with 200-nm intervals was collected. The sample was contrasted with heavy metals and embedded in epoxy resin, and then 250-nm serial sections were collected for EM. Using the fluorescent z stack, the 250-nm EM section bearing the organelles of interest was estimated, imaged, and correlated with the FM data. Figure 5 shows a differential interference contrast (DIC) image from a selected cell (Figure 5B) overlayed with the fluorescence max intensity projection from endocytosed fBSA-Au^5 (Figures 5B and 5C) and a low-magnification overview of the corresponding 250-nm EM section (Figure 5D). We selected perinuclear regions for FM-EM correlation because these are relatively thick and best demonstrate the 3D cellular distribution of endocytosed fBSA-Au^5. We used the information from the FM z stack to assess the distance between an ROI and the bottom of the coverslip. This guided us to select the correct section to acquire high-resolution tomograms of the fBSA-Au^5 containing compartments (the method is outlined in Figure 5A).

In the tomograms, we could easily distinguish the individual organelles selected in FM (Figures 5E and 5F). Similar to the previous examples, the correlation accuracy of this approach ranged between 60 and 200 nm in 2D (determined using ec-CLEM) and was limited by the EM section thickness (250 nm) in z. Most fluorescent spots correlated by ET were endolysosomal compartments containing clusters of gold particles (Figures 5G and 5H). We also found faint fluorescent spots that correlated with endosomal organelles containing only a few gold particles (Figure 5H, organelle 3). This highlights the strong signal of the fBSA-Au^5 probe. We conclude that endocytosed fBSA-Au is highly suitable for resin-based 3D CLEM approaches, first by providing the information required to select the correct Z layer for ET imaging and second by using fBSA-Au as fiducial marker to provide high correlation accuracy in 3D.

(C) Magnified crop from (A), showing the ROI selected for ET. Numbers refer to the same spots as shown in (E) and (F).
(D) Magnification of the ROI with the fluorescence information overlaid. fBSA-Au^5 fluorescence strictly corresponds to fBSA-Au^5 gold particles.
(E) Virtual slice from the tomogram, highlighting selected organelles.
(F) Magnification of selected organelles. (1) shows LEs and LYs with large amounts of fBSA-Au^5 gold particles (white arrows) and minimal LAMP-1 labeling (white arrowheads). (2) shows a LAMP-1-labeled LY devoid of endocytosed fBSA-Au^5. This slice is from the surface of the section, showing LAMP-1 representing gold particles that do not penetrate the section. (3) shows LAMP-1-labeled LY abundantly filled with endocytosed fBSA-Au^5 gold.
(G) Virtual sections through the LY shown in (1) of (F), showing distribution of gold throughout the compartment.
Scale bars: 2 μm (A and B), 500 nm (C–E), 200 nm (F), and 100 nm (G).

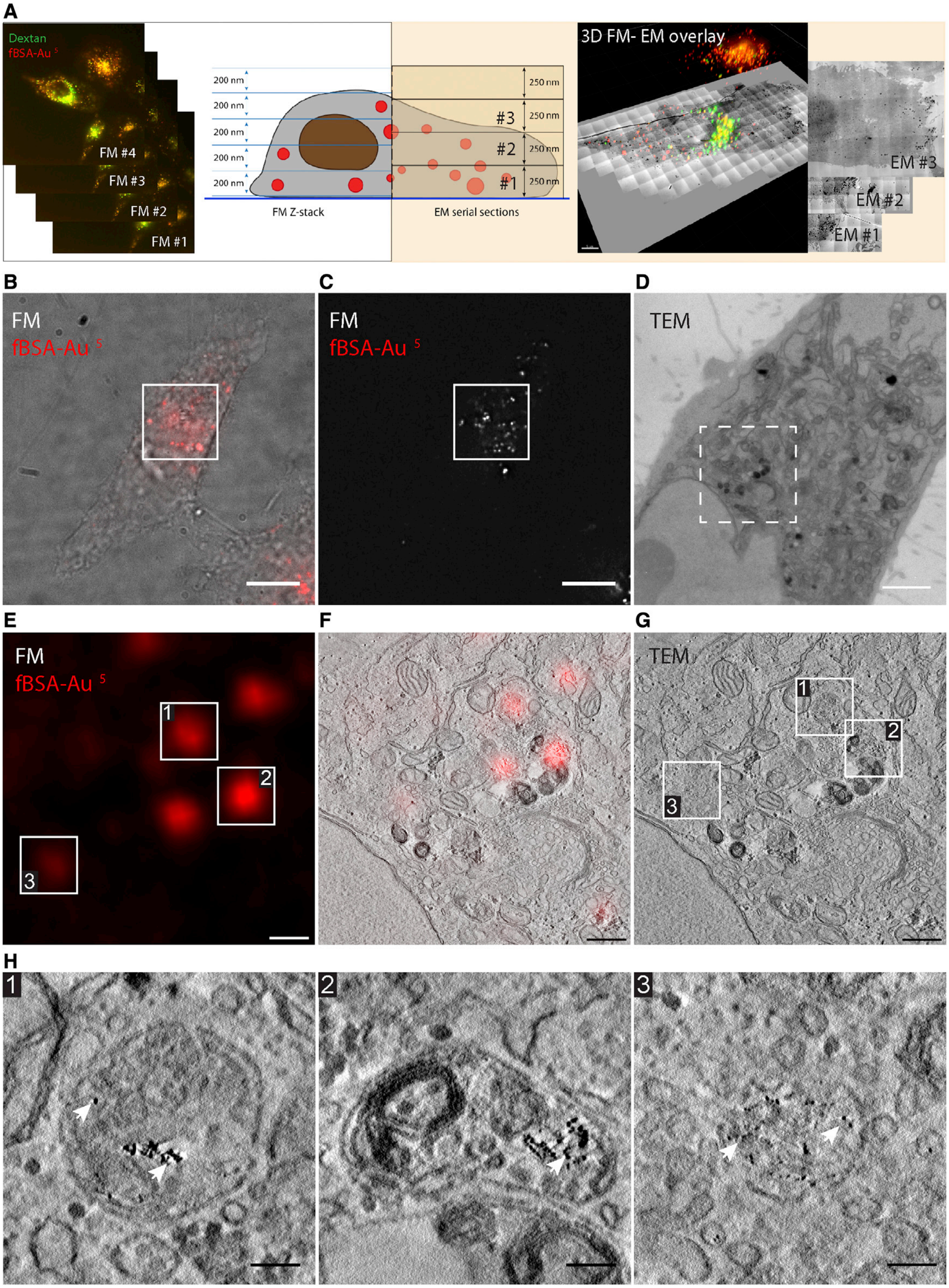

Figure 5. fBSA-Au serves as a bimodal endocytic probe for CLEM using pre-embedding fluorescence and resin sections

HeLa cells were incubated for 3 h with fBSA-Au^5.

(A) Schematic of the imaging strategy employed. A fluorescent z stack with 200-nm intervals is collected after fixation but prior to resin embedding. After resin embedding, 250-nm-thick sections were cut for ET. The depth (z plane) bearing the organelles of interest was estimated based on the fluorescent z stack, and the corresponding section was imaged in ET.

(legend continued on next page)

fBSA-Au as a fiducial marker to mark endolysosomes for cryo-ET

Cellular cryo-ET is an emerging technique for determining the 3D structures of molecules with subnanometer resolution (Beck and Baumeister, 2016). Vitrification of cells keeps molecules in their near native state, and cryo-ET provides high resolution *in situ* images of molecules in the context of the cell. However, the lack of efficient labels for cryo-ET and the low level of contrast makes it challenging to select subcellular ROIs for cryo-ET image acquisition (Arnold et al., 2016; Schorb et al., 2017). Cryo-FM screening prior to cryo-ET is a promising way for identification of ROIs, which can then be re-located and targeted for imaging by cryo-ET (Sun et al., 2019). We postulated that fBSA-Au would be a highly suitable tool to mark endolysosomal organelles by cryo-FM and serve as tool to pre-identify and select regions to collect tomograms by cryo-ET.

For these experiments, we used cultured differentiated neurons (dorsal root ganglion [DRG] neurons) because the less than 500-nm thin axonal projections (transparent for the electron beam) allow direct cryo-ET without the need to prepare lamella (Foster et al., 2021) (Figure 6). We incubated DRGs for 2 h with fBSA-Au^5 and then performed the required washing and vitrification steps (see STAR Methods for the detailed procedure). By cryo-FM, the red fluorescence of fBSA-Au^5 was visible in a thin region of the axon as a collection of small fBSA-Au^5 puncta (Figures 6A and 6B, yellow insets and arrows). There were also some bright fluorescent patches on the empty grid surface (white arrowheads in Figure 6A), which likely correspond to clusters of fBSA-Au^5 adhering to the laminin coat required for neuronal growth because no such patches were observed on glass or uncoated or fibronectin-coated grids. We then used the presence of axonal fBSA-Au^5 labeling to navigate to the axonal regions using TEM and collect cryo-ET data. The reconstructed tomograms of similar ROIs showed various endolysosomal compartments of which approximately half contained fBSA-Au^5. The high contrast of the fBSA-Au particles allowed their unambiguous identification using cryo-ET. The gold appeared as single particles in small vesicles (Figure 6C; Video S2), tubules, and early endosomes (Figures 6D and 6E) and was more clustered in later endolysosomal organelles (Figures 6F and 6G; Videos S3 and S4). These data show that endocytosed fBSA-Au is visible in cryo-FM and cryo-ET and can be applied to mark ROIs for imaging by cryo-ET.

fBSA-Au as a fiducial marker for lamella preparation for cryo-ET

Unlike axons, most biological samples are too thick to perform direct cryo-ET. Instead, 100- to 200-nm lamellae need to be prepared to enable cryo-ET imaging (Rigort et al., 2012). For this procedure, cryo-preserved cells are milled in a cryo-FIB-SEM to create lamellae suitable for cryo-ET imaging. Cryo-FIB milling is the most effective thinning method to date, but imaging of specific biomolecular processes requires localization of ROIs that measure ~1 μm laterally and 200–300 nm in z in larger cells. Especially for rare cellular events or structures, there is no routine method assuring that the structure of interest is present in the prepared lamella. Cryo-FM is used to identify an ROI for cryo-ET. In general, fluorescently labeled cells (e.g., expressing a GFP-tagged protein) are cultured on TEM grids, vitrified, imaged by cryo-FM, and transferred to a cryo-FIB-SEM, where the position for lamellae milling is selected in the x-y plane (laterally) based on the cryo-FM data, and the z position (axially) is estimated to the best accuracy allowed by the FM data. When processed, the lamellae are transferred for cryo-TEM imaging. This approach maximizes targeting of the intended ROI and reduces imaging of areas that do not contain structures of interest (Arnold et al., 2016; Gorelick et al., 2019), increasing the success rate of the method while reducing beamtime. Fiducial markers with a homogeneous 3D distribution throughout the intracellular volume are a highly powerful tool to mark ROIs for cryo-ET. Hence, as a final application, we here test the performance of fBSA-Au in a cryo-ET CLEM workflow, using the fBSA-Au fiducial particles to target lamella preparation and identify targets in the prepared lamellae.

Human bone osteosarcoma epithelial (U2OS) cells were cultured on TEM grids treated with Dynabeads (1-μm-sized, traditionally used fiducials), incubated with fBSA-Au^5 particles for 3 h, and vitrified. A fluorescent z stack showing the position of the Dynabeads (green channel) and fBSA-Au^5 particles (red channel) was collected in a spinning-disk FM equipped with a cryo-module (see Video S5 for the z stack of the cell shown in Figure 7). A maximum-intensity projection of the fBSA-Au^5 particles present in the z stack is shown in Figure 7A. After 3D cryo-FM imaging, the grids were loaded into the cryo-FIB-SEM. First, the FM and SEM overview images were correlated by the distribution of the Dynabeads, providing the x-y-z alignment prior to milling (Figure 7B). Then, using the fluorescent z stack from the fBSA-Au^5 particles (Figure 7D), a lamella (z) position was selected and prepared as described previously (Wagner et al., 2020). The SEM image of the prepared lamella is shown in Figure 7C, overlaid with the fluorescence of the fBSA-Au^5 particles. The prepared lamella was then transferred and imaged using 200-kV cryo-EM. The lamella overview images from cryo-EM were correlated with the cryo-FM data showing the localization of the fBSA-Au^5 particles (overlay shown in Figure 7E). We then recorded high-magnification images (Figures 7G and 7H)

(B) DIC image of a cell with fluorescence of fBSA-Au^5 overlayed in red.
(C) Fluorescence signal of fBSA-Au^5 shown in (A). The ROI for CLEM is highlighted with a white box.
(D) TEM micrograph of a 250-nm-thick section showing the ROIs from (B) and (C). The ROI for ET is indicated by the dashed white box.
(E) Fluorescence signal corresponding to the ET ROI with numbered spots of interest.
(F) Virtual slice from the tomogram, overlaid with fluorescence data.
(G) Virtual slice from the tomogram, showing the 3 selected organelles.
(H) Magnified virtual slices of the selected organelles containing fBSA-Au^5, visible by the 5-nm gold particles (arrows). Organelles 1 and 2 are late endolysosomes, and organelle 3 is an LE.
Scale bars: 10 μm (B and C), 2 μm (D), 500 nm (E–G), and 100 nm (H).

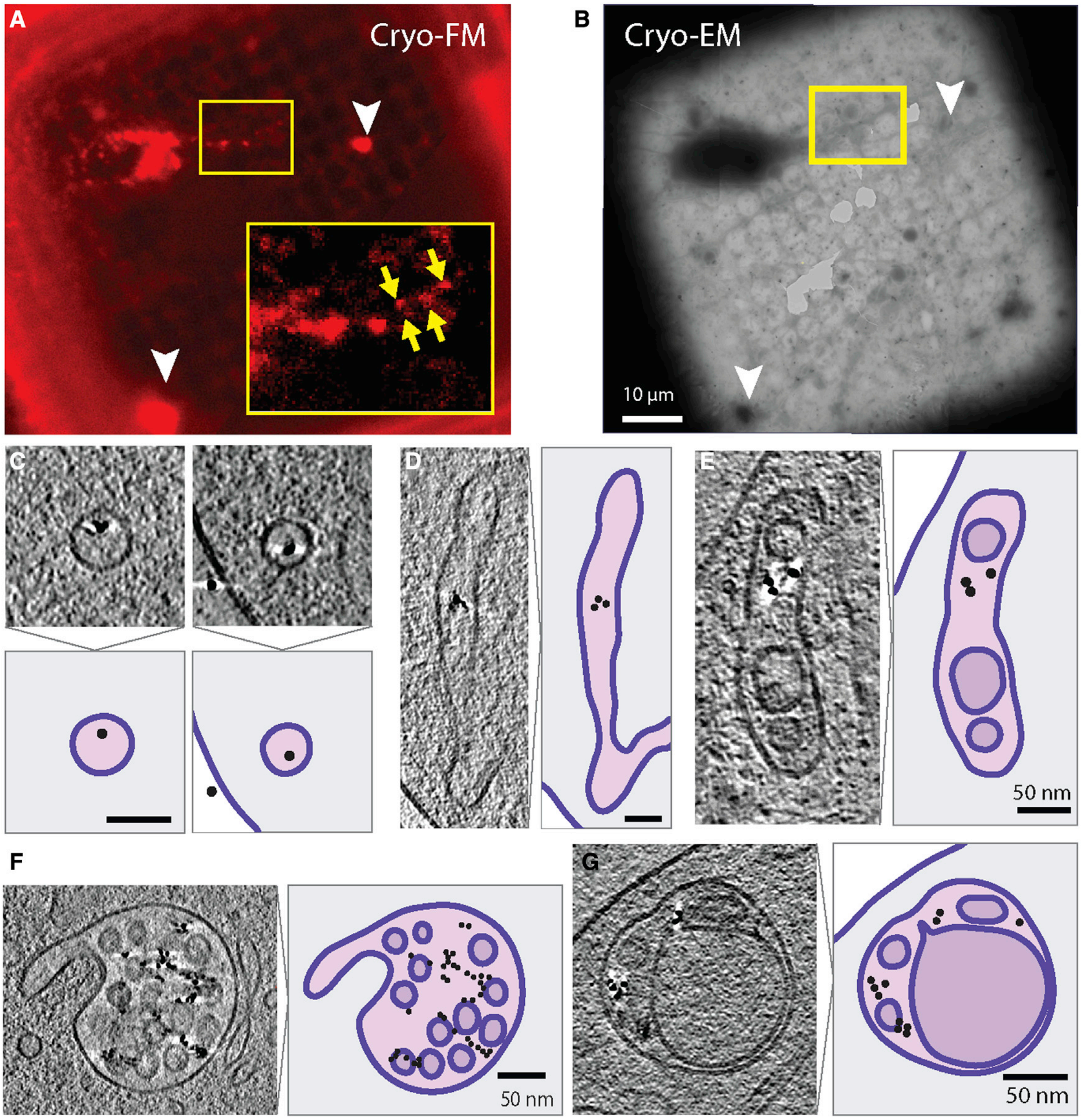

Figure 6. fBSA-Au as a bimodal endocytic probe for cryo-ET

(A and B) Cryo-FM (A) and cryo-EM (B) images of neurons grown on EM grids. The red signal in cryo-FM originates from the Alexa 555 groups of fBSA-Au^5. Yellow arrows point to less intense fluorescent spots corresponding to small endolysosomal organelles in the thin parts of axons. White arrowheads point to dense clusters of fBSA-Au^5 adhering to the laminin-coated grid.

(C) Small vesicles in cryo-ETs of DRG neurons containing fBSA-Au^5 gold particles. A model for each example is shown below, with the lipid bilayer shown in purple, the vesicle lumen in light pink, and fBSA-Au^5 as a black circle. The neuron cytoplasm is shown in gray, and the region outside of the cell is white.

(D and E) As in (C) for tubular structures (D) and as in (C) and (D) for an early endosome (E). The lumen of the internal vesicle is shown in light purple.

(F) As in (C) and (E) for a multi-vesicular body.

(G) As in (F) and (C) for an LE/LY.

Scale bars, 10 μm (A and B) and 50 μm (C–G).

of the correlated organelles (indicated with green and blue squares in Figures 7E and 7F), showing endocytosed fBSA-Au^5 particles in late endosomes and lysosomes. The correlation accuracy between cryo-FM and cryo-EM was approximately 100 nm (defined by the organelle size). We managed to follow a single fluorescent compartment (indicated by a red arrow) throughout the complete cryo-CLEM procedure and recorded the tomogram (Video S6, corresponding to the red arrow in Figure 7).

These data show that endocytosed fBSA-Au can be used as a 3D fiducial marker in a cryo-CLEM workflow. The 3D distribution of the particles in the endolysosomal system makes them very suitable to select regions for lamella preparation based on the cryo-FM signal. The strong contrast of the particles in

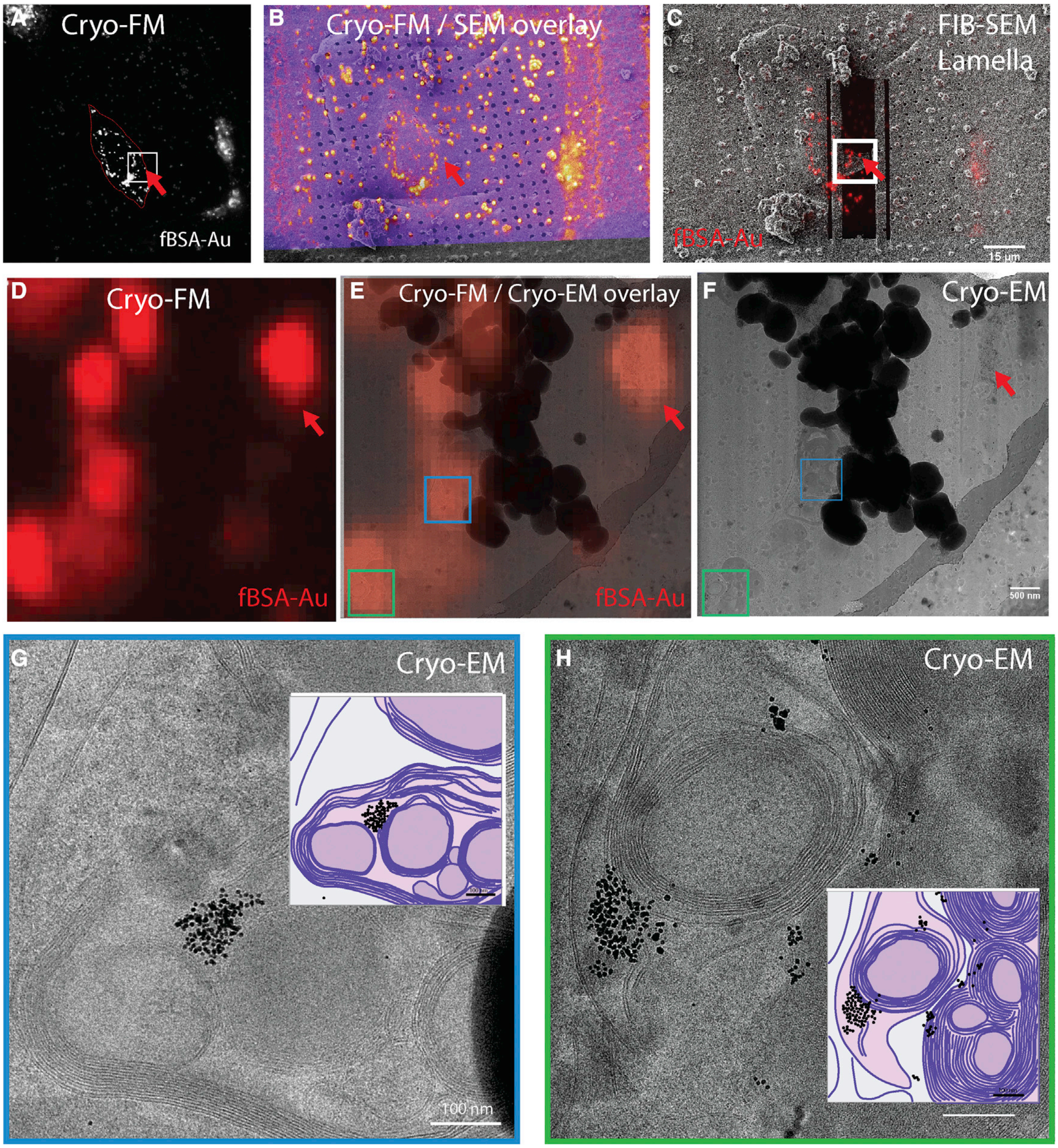

Figure 7. fBSA-Au as a fiducial marker for cryo-CLEM

(A) Red channel maximum intensity projection of the cryo-FM z stack of the selected U2OS cell. The red signal in cryo-FM originates from the Alexa 555 groups of fBSA-Au^5.

(B) Green channel cryo-FM image (Dynabeads) overlayed onto the SEM overview.

(C) Magnified cryo-FM image of the ROI, showing the organelles bearing fBSA-Au^5 selected for lamella preparation. The ROI is also depicted with a white square in (A).

(D) Post-milling SEM image of the prepared lamella overlayed with the corresponding individual z stack image of the FM data.

(E) Overlay of the cryo-FM signal of fBSA-Au^5 in the corresponding organelles in the lamella and cryo-TEM images.

(F) Overview cryo-TEM image of the lamella. Organelles imaged with higher resolution are depicted with green and blue squares and are shown in (G) and (H), respectively.

(G) A LY bearing fBSA-Au^5 in its lumen.

(H) Another LY bearing fBSA-Au^5. A model for each organelle is shown as an inset, with the lipid bilayer shown in purple, the lumen in light pink, and fBSA-Au^5 as black circles.

Scale bars: 15 µm (B and D), 500 nm (E and F), and 100 nm (G and H).

cryo-TEM makes their visualization easy and increases the accuracy in tomogram reconstruction to visualize fine structural elements. For example, reconstructions of fBSA-Au^5 endosomes readily show contact sites between endosomes and the cytoskeleton (Figure S4; Video S7).

DISCUSSION

Here, we introduce fBSA-Au, a conjugate of Alexa Fluor-labeled BSA and 5- or 10-nm sized colloidal gold particles and describe its application as a bimodal endocytic probe and 3D fiducial marker. Endocytic markers are an attractive solution for correlative fiducials because they efficiently distribute throughout cells, resulting in a well-defined 3D pattern of landmarks usable for correlation. We demonstrate that fBSA-Au^5 and fBSA-Au^{10} are stable in solution, efficiently endocytosed, non-toxic, and brightly fluorescent and have good electron contrast for detection in TEM. The probe is versatile because it can be synthesized with differently sized gold particles and distinct fluorophores. We successfully generated fBSA-Au^5 and fBSA-Au^{10} particles with Alexa 488, Alexa 555, Alexa 647, Texas red, and tetramethylrhodamine (TRITC) fluorophores. We show that endocytosed fBSA-Au is taken up effectively by cells, colocalizes with markers of early and late endosomal compartments (EEA1, CD63, and LAMP1), and reaches the same population of organelles as established endocytic tracers, such as dextran or fluorescent BSA.

The properties of fBSA-Au render it broadly applicable for 2D and 3D CLEM and compatible with resin EM, cryosectioning, and cryo-EM approaches. We show that fBSA-Au provides ample fluorescence signal for imaging in intact cells under live and fixed conditions because of build-up of multiple fBSA-Au particles in the confined space of endosomes and lysosomes. In addition, fBSA-Au is readily detectable even in FM of ultrathin (70 nm) Tokuyasu cryosections. The high electron contrast and uniformly sized gold particles make them easily visible in TEM and provide reliable reference points to correlate data from FM and EM in 2D and 3D. Because the gold particles in fBSA-Au are of well-defined size, their use is compatible with immunogold labeling, which is especially valuable when using cryosection CLEM. We demonstrated use of fBSA-Au in TEM-based CLEM, but we envision that fBSA-Au is also compatible with SEM-based CLEM approaches, using 3D EM techniques like FIB-SEM, serial blockface (SBF) SEM, and array tomography. SEM-based 3D methods enable examination of larger volumes than ET but with less resolution (Peddie and Collinson, 2014), which likely necessitates use of fBSA-Au^{10}. The excellent visibility in EM ensures compatibility with current and future automated CLEM procedures and registration software (Sjollema and Giepmans, 2016; Paul-Gilloteaux et al., 2017; Mohammadian et al., 2019). Combining the uncompromised FM and EM properties, we found that the probe enables easy registration of CLEM data in 2D and 3D applications. The applicability also extends to live-cell CLEM approaches because the endocytosed fiducials are visible during every step of correlative imaging, all the way from live cells to the final tomograms (Figures 2 and 7). Bimodal nanoparticles are a useful tool in cell biology, either as bimodal fiducial for CLEM or as an endocytic probe to mark endolysosomal organelles.

Previously, we and others introduced fluorescently labeled silica nanoparticles as bimodal endocytic tracers and fiducials for FM and EM (Zhang and Hensel, 2013; Fokkema et al., 2018). These particles provide easy correlation and perform well as extracellular fiducials for CLEM. Their distinct shape with a well-defined gold core also enables a Gaussian fitting step to obtain better precision between single particles and fluorescence. However, their relatively large size (~100 nm) limits the efficiency of endocytosis and may obscure morphological features in endosomes, hampering their ultrastructural identification. The fBSA-Au probe presented here is significantly smaller (5- to 15-nm diameter versus 60–100 nm for silica particles), which allows higher levels of endocytosis while the visibility of morphological features in endosomes is retained. Quantum dots (Giepmans et al., 2005; Peckys et al., 2014; Liv et al., 2015) and nanodiamonds (Han et al., 2019; Prabhakar et al., 2020) are another type of bimodal nanoparticles used in cell biology and CLEM approaches. Like fBSA-Au, they can be functionalized with physiologically relevant molecules and are small in size (5–30 nm). However, their lower electron density makes them poorly visible in electron micrographs, in contrast to the excellent EM visibility of fBSA-Au. Quantum dots remain invisible in cryosections because of the negative contrasting protocol.

A limitation in development of bimodal fiducials is that fluorophores can be quenched when they are in close proximity to colloidal heavy metal particles (Kandela et al., 2003; Kandela and Albrecht, 2007; Miles et al., 2017). Usually, spacers are incorporated to prevent this quenching, especially when larger-sized metal particles are used, but these probes still suffer from a limited fluorescence signal. Because of the synthesis strategy of fBSA-Au, where Alexa-labeled BSA proteins are bound to colloidal gold particles, we achieved a high fluorescence signal despite using relatively large gold particles. In our case, the bulk of the BSA molecules provides enough space between the gold and the fluorophores to retain sufficient fluorescence. Commercially available BSA-Alexa 555 is labeled with Alexa 555 at a 5:1 molar density (5 mol of dye per 1 mol protein). This high labeling density combined with the bulk of BSA should provide a sufficiently large fraction of unquenched fluorophores visible in FM.

One of the challenges with bimodal probes is to ensure colocalization of the fluorescence signal and the electron-dense particle (Van E. et al., 2017; Miles et al., 2017). For fBSA-Au^5, the challenge primarily lies in degradation of BSA as it is transported to lysosomes, the enzymatically active endpoint of the endocytic pathway. Degradation of BSA will lead to a dissociation of the Alexa label and gold particles, which may lead to labeling discrepancies in correlative approaches. In previous studies, degradation of BSA was seen by aggregation and clustering of gold particles in lysosomes (Bright et al., 1997; Pols et al., 2013). Similar clustering was observed in lysosomes in the experiments here, indicating degradation of BSA-Alexa 555 (Figures 3 and 4). Our CLEM experiments show no labeling discrepancies between FM and EM; gold particles were seen in every fluorescently labeled compartment, and, vice versa, fluorescence was detected in all compartments containing gold colloids. This overlap confirms that, at least within a period of 3 h of uptake, the localization and fluorescence of the Alexa dye is

retained in endocytic compartments, even after degradation of BSA, and throughout the process of fixation, sectioning, and labeling.

In cryo-EM, accurate selection of ROIs for cryo-ET is of extreme importance because of the fragile nature of frozen hydrated material (Villa et al., 2013). Use of fluorescence to determine ROIs prior to imaging is rapidly gaining traction thanks to maturing cryo-FM setups (Schwartz et al., 2007; van Driel et al., 2009; Schellenberger et al., 2013; Kaufmann et al., 2014b). Fluorophores generally retain their fluorescence and exhibit reduced bleaching at cryogenic temperatures (Weisenburger et al., 2013; Kaufmann et al., 2014a, 2014b). We have shown that fBSA-Au is compatible with cryo-FM and cryo-EM of vitrified material. By using the 3D distribution of endocytosed fBSA-Au, we can select ROIs by cryo-FM, use this fluorescence to identify where to make lamella in the cryo-FIB-SEM, and locate the targeted organelles within the lamella during subsequent imaging with cryo-ET. fBSA-Au can be a highly suitable fiducial marker, especially in integrated FM in FIB-SEM systems, where the ROI (e.g., lamella) identified by FM can be directly prepared for cryo-ET (Smeets, 2020; Timmermans et al., 2016; Loginov et al., 2022). An additional benefit of endocytosed nanogold fiducials for cryo-ET is accurate tracking during tilt series acquisition and improved tilt-series alignment for image reconstruction, as reported by others (Berger et al., 2021).

We conclude that fBSA-Au is a powerful and easy-to-use 3D fiducial marker that can be used in an array of CLEM applications because it is stable, efficiently endocytosed, and compatible with a variety of established FM and EM techniques. fBSA-Au provides high correlation accuracy in 2D and 3D CLEM applications and is especially suited to address questions at the subcellular level requiring high correlation efficiency.

Limitations of the study

The examples provided here show CLEM of endolysosomal organelles, but the bimodal visibility of fBSA-Au is also highly usable for CLEM studies of other cellular structures. Endosomes containing fBSA-Au can be used as 3D reference points with a unique spatial distribution to register FM data from other structures of interest beyond the endolysosomal system to their corresponding ultrastructure with a correlation precision comparable with organelles bearing fBSA-Au particles (Figures 3 and 4). Because nearly all cell types show a significant level of endocytosis, with the exception of erythrocytes, use of endocytosed fBSA-Au is widely applicable to a large variety of cellular and tissue models. In cryo-ET applications, a possible limitation can arise from the weak phase approximation expected from tomogram reconstruction, where the averaging of structures close to the dark Au particles can be perturbed. However, this is only relevant for averaging structures in the endolysosomal lumen, where the Au particles are localized. Imaging closer to focus and minimizing the non-suitable area with a dense Au particle presence would be a solution. Finally, in CLEM applications where EM does not provide enough resolution to visualize 5-nm or 10-nm Au particles, bigger fiducial probes, like the fluorescently labeled silica nanoparticles we have reported previously (Fokkema et al., 2018), might be preferable.

STAR★METHODS

Detailed methods are provided in the online version of this paper and include the following:

- KEY RESOURCES TABLE
- RESOURCE AVAILABILITY
  - Lead contact
  - Materials availability
  - Data and code availability
- EXPERIMENTAL MODEL AND SUBJECT DETAILS
  - Cell lines and culture
- METHOD DETAILS
  - fBSA-Au^5 and fBSA-Au^{10} complex synthesis
  - Immunofluorescence labelling and imaging of endocytosed fBSA-Au
  - Correlative microscopy of resin-embedded samples
  - Sample preparation and light microscopic imaging of Tokuyasu cryosections
  - Electron microscopy of resin sections and Tokuyasu cryosections
  - Cryo-FM and Cryo-EM of DRG neurons
  - Cryo-FM and Cryo-EM of U2OS cells
  - Tomogram reconstruction
  - Correlation of light and electron microscopy images
- QUANTIFICATION AND STATISTICAL ANALYSIS
  - Size distribution analysis of fBSA-Au^5 and fBSA-Au^{10}
  - Colocalization analysis
  - Correlation accuracy of FM and EM images

SUPPLEMENTAL INFORMATION

Supplemental information can be found online at https://doi.org/10.1016/j.crmeth.2022.100220.

ACKNOWLEDGMENTS

We acknowledge funding from the Netherlands Organisation for Scientific Research (NWO), Stichting voor de Technische Wetenschappen (Technology Foundation STW), grant 12715 (to H.G. and J.K.) and the Netherlands Organisation for Health Research and Development (ZonMW) grant 40-00812-98-16006 (to J.K.). The microscopy infrastructure in this work is partially subsidized by the Roadmap for Large-Scale Research Infrastructure (NEMI) of Netherlands Organisation for Scientific Research (grant number 184.034.014) (to J.K.). The Cell Microscopy Core of UMC Utrecht is part of the Dutch Correlative Light Electron Microscopy node of EuroBioImaging.

AUTHOR CONTRIBUTIONS

N.L., J.F., G.P., H.C.G., and J.K. designed the study. G.P. conceived the bimodal probes. T.V., V.O., and S.v.D. synthesized, optimized, and characterized the endocytic fiducials. J.F., T.V., L.Y., and C.d.H. optimized the cellular uptake and imaging of endocytic fiducials and analyzed the data with N.L. J.F. performed 2D on-section CLEM and 3D correlative FM-ET studies. H.E.F. performed cryo-ET experiments with neurons. L.d.J. and S.H. performed cryo-CLEM experiments of U2OS cells. J.F., L.d.J., and H.E.F. performed image correlation and segmentation and prepared the corresponding figures with N.L. W.H.M., W.L., S.H., A.P.C., F.F., H.C.G., J.K., and N.L. supervised the study. J.F. and N.L. wrote the paper with input from all authors. All authors reviewed the manuscript.

CellPress
OPEN ACCESS

DECLARATION OF INTERESTS

The endocytic fBSA-Au^5 and fBSA-Au^{10} fiducials reported here are available in the product listing of Cell Microscopy Core, UMC Utrecht.

Received: May 16, 2021
Revised: February 4, 2022
Accepted: April 26, 2022
Published: May 16, 2022

REFERENCES

Arnold, J., Mahamid, J., Lucic, V., de Marco, A., Fernandez, J.J., Laugks, T., Mayer, T., Hyman, A., Baumeister, W., and Plitzko, J. (2016). Site-specific cryo-focused ion beam sample preparation guided by 3D correlative microscopy. Biophys. J. *110*, 860–869. https://doi.org/10.1016/j.bpj.2015.10.053.

Beck, M., and Baumeister, W. (2016). Cryo-electron tomography: can it reveal the molecular sociology of cells in atomic detail? Trends Cell Biol., 825–837. https://doi.org/10.1016/j.tcb.2016.08.006.

Berger, C., Ravelli, Raimond B.G., López-Iglesias, C., and Peters, P.J. (2021). Endocytosed nanogold fiducials for improved in-situ cryo–electron tomography tilt-series alignment. J. Struct. Biol. *213*, 107698. https://doi.org/10.1016/j.jsb.2021.107698.

Bishop, D., Nikic, I., Brinkoetter, M., Knecht, S., Potz, S., Kerschensteiner, M., and Misgeld, T. (2011). Near-infrared branding efficiently correlates light and electron microscopy. Nat. Methods *8*, 568–570. https://doi.org/10.1038/nmeth.1622.

Bright, N.A., Reaves, B., Mullock, B., and Luzio, J. (1997). Dense core lysosomes can fuse with late endosomes and are re-formed from the resultant hybrid organelles. J. Cell Sci. *110*, 2027–2040. https://doi.org/10.1242/jcs.110.17.2027.

Bushby, A.J., Mariggi, G., Armer, H.E., and Collinson, L.M. (2012). Correlative light and volume electron microscopy. Methods Cell Biol. *111*, 357–382. https://doi.org/10.1016/B978-0-12-416026-2.00018-2.

Chithrani, B.D., Ghazani, A.A., and Chan, W.C.W. (2006). Determining the size and shape dependence of gold nanoparticle uptake into mammalian cells. Nano Lett. *6*, 662–668. https://doi.org/10.1021/nl052396o.

Connor, E.E., Mwamuka, J., Gole, A., Murphy, C., and Wyatt, M. (2005). Gold nanoparticles are taken up by human cells but do not cause acute cytotoxicity. Small *1*, 325–327. https://doi.org/10.1002/smll.200400093.

De Chaumont, F., Dallongeville, S., Chenouard, N., Hervé, N., Pop, S., Provoost, T., Meas-Yedid, V., Pankajakshan, P., Lecomte, T., Le Montagner, Y., et al. (2012). Icy: an open bioimage informatics platform for extended reproducible research. Nat. Methods, 690–696. https://doi.org/10.1038/nmeth.2075.

Deerinck, T.J., Martone, M.E., Lev-Ram, V., Green, D.P., Tsien, R.Y., Spector, D.L., Huang, S., Ellisman, M.H., et al. (1994). Fluorescence photooxidation with eosin: a method for high resolution immunolocalization and in situ hybridization detection for light and electron microscopy. J. Cell Biol. *126*, 901–910. https://doi.org/10.1083/jcb.126.4.901.

Fermie, J., Liv, N., ten Brink, C., van Donselaar, E.G., Muller, W.H., Schieber, N.L., Schwab, Y., Gerritsen, H.C., and Klumperman, J. (2018). Single organelle dynamics linked to 3D structure by correlative live-cell imaging and 3D electron microscopy. Traffic *19*, 354–369. https://doi.org/10.1111/tra.12557.

Fokkema, J., Fermie, J., Liv, N., van den, Heuvel, Konings, T.O.M., Dave, J., Blab, G.A., Meijerink, A., Klumperman, J., and Gerritsen, H.C. (2018). Fluorescently labelled silica coated gold nanoparticles as fiducial markers for correlative light and electron microscopy. Sci. Rep. *8*, 13625. https://doi.org/10.1038/s41598-018-31836-1.

Foster, H.E., Ventura Santos, C., and Carter, A.P. (2021). A cryo-ET survey of intracellular compartments within mammalian axons. Preprint at bioRxiv. https://doi.org/10.1101/2021.03.29.437454.

Foster, H.E., Ventura Santos, C., and Carter, A.P. (2022). A cryo-ET survey of microtubules and intracellular compartments in mammalian axons. J. Cell Biol. *221*. https://doi.org/10.1083/jcb.202103154.

Franke, C., Repnik, U., Segeletz, S., Brouilly, N., Kalaidzidis, Y., Verbavatz, J., and Zerial, M. (2019). Correlative single-molecule localization microscopy and electron tomography reveals endosome nanoscale domains. Traffic *20*, 601–617. https://doi.org/10.1111/tra.12671.

Gaietta, G.M., Deerinck, T.J., and Ellisman, M.H. (2011). Labeling tetracysteine-tagged proteins with biarsenical dyes for live cell imaging. Cold Spring Harb. Protoc. *2011*, pdb.prot5547–47. https://doi.org/10.1101/pdb.prot5547.

Geoffroy, J.S., and Becker, R.P. (1984). Endocytosis by endothelial phagocytes: uptake of bovine serum albumin-gold conjugates in bone marrow. J. Ultrasruct. Res. *89*, 223–239. https://doi.org/10.1016/S0022-5320(84)80039-8.

Geuze, H.J., Slot, J.W., van der Ley, P.A., and Scheffer, R.C. (1981). Use of colloidal gold particles in double-labeling immunoelectron microscopy of ultrathin frozen tissue sections. J. Cell Biol. *89*, 653–665. http://www.ncbi.nlm.nih.gov/pubmed/6166621.

Giepmans, B.N.G., Deerinck, T.J., Smarr, B.L., Jones, Y.Z., and Ellisman, M.H. (2005). Correlated light and electron microscopic imaging of multiple endogenous proteins using Quantum dots. Nat. Methods *2*, 743–749. https://doi.org/10.1038/nmeth791.

Gorelick, S., Buckley, G., Gervinskas, G., Johnson, T.K., Handley, A., Caggiano, M.P., Whisstock, J.C., Pocock, R., and de Marco, A. (2019). PIE-scope, integrated cryo-correlative light and FIB/SEM microscopy. eLife *8*. https://doi.org/10.7554/eLife.45919.

Hagen, W.J.H., Wan, W., and Briggs, J.A.G. (2017). Implementation of a cryo-electron tomography tilt-scheme optimized for high resolution subtomogram averaging. J. Struct. Biol. *197*, 191–198. https://doi.org/10.1016/j.jsb.2016.06.007.

Han, S., Raabe, M., Hodgson, L., Mantell, J., Verkade, P., Lasser, T., Landfester, K., Weil, T., and Lieberwirth, I. (2019). High-contrast imaging of nanodiamonds in cells by energy filtered and correlative light-electron microscopy: toward a quantitative nanoparticle-cell analysis. Nano Lett. *19*, 2178–2185. https://doi.org/10.1021/acs.nanolett.9b00752.

Hanaki, K.I., Momo, A., Oku, T., Komoto, A., Maenosono, S., Yamaguchi, Y., and Yamamoto, K. (2003). Semiconductor quantum dot/albumin complex is a long-life and highly photostable endosome marker. Biochem. Biophys. Res. Commun. *302*, 496–501. https://doi.org/10.1016/S0006-291X(03)00211-0.

Hoffman, D.P., Shtengel, G., Xu, C.S., Campbell, K.R., Freeman, M., Wang, L., Milkie, D.E., Pasolli, H.A., Iyer, N., Bogovic, J.A., et al. (2020). Correlative three-dimensional super-resolution and block-face electron microscopy of whole vitreously frozen cells. Science *367*, eaaz5357. https://doi.org/10.1126/science.aaz5357.

Horisberger, M., and Rosset, J. (1977). Colloidal gold, a useful marker for transmission and scanning electron microscopy. J. Histochem. Cytochem. *25*, 295–305. https://doi.org/10.1177/25.4.323352.

Jonker, C.T.H., Galmes, R., Veenendaal, T., ten Brink, C., van der Welle, R.E.N., Liv, N., de Rooij, J., Peden, A.A., van der Sluijs, P., Margadant, C., and Klumperman, J. (2018). Vps3 and Vps8 control integrin trafficking from early to recycling endosomes and regulate integrin-dependent functions. Nat. Commun. *9*, 792. https://doi.org/10.1038/s41467-018-03226-8.

Kandela, I.K., and Albrecht, R.M. (2007). Fluorescence quenching by colloidal heavy metals nanoparticles: implications for correlative fluorescence and electron microscopy studies. Scanning *29*, 152–161. https://doi.org/10.1002/sca.20055.

Kandela, I., Meyer, D., Oshel, P., Rosa-Molinar, E., and Albrecht, R.M. (2003). Fluorescence quenching by colloidal heavy metals: implications for correlative fluorescence and electron microscopy studies. Microsc. Microanal. *9*, 1194–1195. https://doi.org/10.1017/s1431927603445972.

Karreman, M.A., Mercier, L., Schieber, N.L., Solecki, G., Allio, G., Winkler, F., Ruthensteiner, B., Goetz, J.G., and Schwab, Y. (2016). Fast and precise

targeting of single tumor cells in vivo by multimodal correlative microscopy. J. Cell Sci. *129*, 444–456. https://doi.org/10.1242/jcs.181842.

Kaufmann, R., Schellenberger, P., Seiradake, E., Dobbie, I.M., Jones, E.Y., Davis, I., Hagen, C., and Grunewald, K. (2014a). Super-resolution microscopy using standard fluorescent proteins in intact cells under cryo-conditions. Nano Lett. *14*, 4171–4175. https://doi.org/10.1021/nl501870p.

Kaufmann, R., Hagen, C., and Grünewald, K. (2014b). Fluorescence cryo-microscopy: current challenges and prospects. Curr. Opin. Chem. Biol. *20*, 86–91. https://doi.org/10.1016/j.cbpa.2014.05.007.

Kopek, B.G., Shtengel, G., Xu, C.S., Clayton, D.A., and Hess, H.F. (2012). Correlative 3D superresolution fluorescence and electron microscopy reveal the relationship of mitochondrial nucleoids to membranes. Proc. Natl. Acad. Sci. U S A *109*, 6136–6141. https://doi.org/10.1073/pnas.1121558109.

Kremer, J.R., Mastronarde, D.N., and McIntosh, J.R. (1996). Computer visualization of three-dimensional image data using IMOD. J. Struct. Biol. *116*, 71–76. https://doi.org/10.1006/jsbi.1996.0013.

Kukulski, W., Schorb, M., Welsch, S., Picco, A., Kaksonen, M., and Briggs, J.A. (2011). Correlated fluorescence and 3D electron microscopy with high sensitivity and spatial precision. J. Cell Biol. *192*, 111–119. https://doi.org/10.1083/jcb.201009037.

Liv, N., van Oosten Slingeland, D.S.B., Baudoin, J.P., Kruit, P., Piston, D.W., and Hoogenboom, J.P. (2015). Electron microscopy of living cells during in situ fluorescence microscopy. ACS Nano *10*, 265–273. https://doi.org/10.1021/acsnano.5b03970.

Loginov, S., Fermie, J., Fokkema, J., Agronskaia, A.V., Heus, C.D., Blab, G.A., Klumperman, J., Gerritsen, H.C., and Liv, N. (2022). Correlative Organelle Microscopy: Fluorescence Guided Volume Electron Microscopy of Intracellular Processes. Front. Cell Dev. Biol. https://doi.org/10.3389/fcell.2022.829545.

Makarow, M. (1985). Endocytosis in Saccharomyces cerevisiae: internalization of alpha-amylase and fluorescent dextran into cells. EMBO J. *4*, 1861–1866. https://doi.org/10.1002/j.1460-2075.1985.tb03861.x.

Mari, M., Geerts, W.J.C., and Reggiori, F. (2014). Immuno- and correlative light microscopy-electron tomography methods for 3D protein localization in yeast. Traffic *15*, 1164–1178. https://doi.org/10.1111/tra.12192.

Martell, J.D., Deerinck, T.J., Sancak, Y., Poulos, T.L., Mootha, V.K., Sosinsky, G.E., Ellisman, M.H., and Ting, A.Y. (2012). Engineered ascorbate peroxidase as a genetically encoded reporter for electron microscopy. Nat. Biotechnol. *30*, 1143–1148. https://doi.org/10.1038/nbt.2375.

Mastronarde, D.N. (2005). Automated electron microscope tomography using robust prediction of specimen movements. J. Struct. Biol. *152*, 36–51. https://doi.org/10.1016/j.jsb.2005.07.007.

Mastronarde, D. (2018). Advanced Data Acquisition From Electron Microscopes With SerialEM. Microscopy and Microanalysis *24*, 864–865. https://doi.org/10.1017/S1431927618004816.

Mastronarde, D.N., and Held, S.R. (2017). Automated tilt series alignment and tomographic reconstruction in IMOD. J. Struct. Biol. *197*, 102–113. https://doi.org/10.1016/j.jsb.2016.07.011.

Miles, B.T., Greenwood, A.B., Benito-Alifonso, D., Tanner, H., Galan, M.C., Verkade, P., and Gersen, H. (2017). Direct evidence of lack of Colocalisation of fluorescently labelled gold labels used in correlative light electron microscopy. Sci. Rep. *7*, 44666. https://doi.org/10.1038/srep44666.

Mohammadian, S., Fokkema, J., Agronskaia, A.V., Liv, N., de Heus, C., van Donselaar, E., Blab, G.A., Klumperman, J., and Gerritsen, H.C. (2019). High accuracy, fiducial marker-based image registration of correlative microscopy images. Sci. Rep. *9*, 3211. https://doi.org/10.1038/s41598-019-40098-4.

Müller-Reichert, T., Srayko, M., Hyman, A., O'Toole, E.T., and McDonald, K. (2007). Correlative light and electron microscopy of early Caenorhabditis elegans embryos in mitosis. Methods Cell Biol. *2007*, 101–119. https://doi.org/10.1016/S0091-679X(06)79004-5.

Nickell, S., Forster, F., Linaroudis, A., Net, W.D., Beck, F., Hegerl, R., Baumeister, W., and Plitzko, J.M. (2005). TOM software toolbox: acquisition and analysis for electron tomography. J. Struct. Biol. *149*, 227–234. https://doi.org/10.1016/j.jsb.2004.10.006.

Oorschot, V.M.J., Sztal, T.E., Bryson-Richardson, R.J., and Ramm, G. (2014). Immuno correlative light and electron microscopy on tokuyasu cryosections. Methods Cell Biol. https://doi.org/10.1016/B978-0-12-801075-4.00011-2.

Paul-Gilloteaux, P., Heiligenstein, X., Belle, M., Domart, M.C., Larijani, B., Collinson, L., Raposo, G., and Salamero, J. (2017). EC-CLEM: flexible multidimensional registration software for correlative microscopies. Nat. Methods, 102–103. https://doi.org/10.1038/nmeth.4170.

Peckys, D.B., Bandmann, V., and de Jonge, N. (2014). Correlative fluorescence and scanning transmission electron microscopy of quantum dot-labeled proteins on whole cells in liquid. Methods Cell Biol. https://doi.org/10.1016/B978-0-12-801075-4.00014-8.

Peddie, C.J., and Collinson, L.M. (2014). Exploring the third dimension: volume electron microscopy comes of age. Micron *61*, 9–19. https://doi.org/10.1016/j.micron.2014.01.009.

Peddie, C.J., Liv, N., Hoogenboom, J.P., and Collinson, L.M. (2014). Integrated light and scanning electron microscopy of GFP-expressing cells. Methods Cell Biol., 363–389. https://doi.org/10.1016/B978-0-12-801075-4.00017-3.

Polishchuk, R.S., Polishchuk, E.V., Marra, P., Alberti, S., Buccione, R., Luini, A., and Mironov, A.A. (2000). Correlative light-electron microscopy reveals the tubular-saccular ultrastructure of carriers operating between Golgi apparatus and plasma membrane. J. Cell Biol. *148*, 45–58. https://doi.org/10.1083/jcb.148.1.45.

Pols, M.S., ten Brink, C., Gosavi, P., Oorschot, V., and Klumperman, J. (2013). The HOPS proteins hVps41 and hVps39 are required for homotypic and heterotypic late endosome fusion. Traffic *14*, 219–232. https://doi.org/10.1111/tra.12027.

Powell, R.D., Halsey, C.M.R., and Hainfeld, J.F. (1998). Combined fluorescent and gold immunoprobes: reagents and methods for correlative light and electron microscopy. Microsc. Res. Tech. *42*, 2–12. https://doi.org/10.1002/(SICI)1097-0029(19980701)42:1<2::AID-JEMT2>3.0.CO;2-Y.

Prabhakar, N., Belevich, I., Peurla, M., Heiligenstein, X., Chang, H.C., Sahlgren, C., Jokitalo, E., and Rosenholm, J.M. (2020). Cell volume (3D) correlative microscopy facilitated by intracellular fluorescent nanodiamonds as multimodal probes. Nanomaterials *11*, 14. https://doi.org/10.3390/nano11010014.

Rigort, A., Bauerlein, F.J.B., Villa, E., Eibauer, M., Laugks, T., Baumeister, W., and Plitzko, J.M. (2012). Focused ion beam micromachining of eukaryotic cells for cryoelectron tomography. Proc. Natl. Acad. Sci. U S A *109*, 4449–4454. https://doi.org/10.1073/pnas.1201333109.

Roth, J., Bendayan, M., and Orci, L. (1980). FITC-protein A-gold complex for light and electron microscopic immunocytochemistry. J. Histochem. Cytochem. *28*, 55–57. https://doi.org/10.1177/28.1.6153194.

Russell, M.R.G., Lerner, T.R., Lerner, T.R., Lerner, T.R., Pelchen-Matthews, A., Domart, M.C., Durgan, J., Weston, A., Jones, M.L., Peddie, C.J., et al. (2016). 3D correlative light and electron microscopy of cultured cells using serial blockface scanning electron microscopy. J. Cell Sci. jcs.188433. https://doi.org/10.1242/jcs.188433.

Schellenberger, P., Kaufmann, R., Siebert, C.A., Hagen, C., Wodrich, H., and Grunewald, K. (2013). High-precision correlative fluorescence and electron cryo microscopy using two independent alignment markers. Ultramicroscopy *143*, 41–51. https://doi.org/10.1016/j.ultramic.2013.10.011.

Schneider, C.A., Rasband, W.S., and Eliceiri, K.W. (2012). NIH Image to ImageJ: 25 years of image analysis. Nat. Methods *9*, 671–675. https://doi.org/10.1038/nmeth.2089.

Schorb, M., Gaechter, L., Avinoam, O., Sieckmann, F., Clarke, M., Bebeacua, C., Bykov, Y.S., Sonnen, A.F.P., Lihl, R., and Briggs, J.A. (2017). New hardware and workflows for semi-automated correlative cryo-fluorescence and cryo-electron microscopy/tomography. J. Struct. Biol. *197*, 83–93. https://doi.org/10.1016/j.jsb.2016.06.020.

Schwartz, C.L., Sarbash, V.I., Ataullakhanov, F.I., Mcintosh, J.R., and Nicastro, D. (2007). Cryo-fluorescence microscopy facilitates correlations between

CellPress
OPEN ACCESS

light and cryo-electron microscopy and reduces the rate of photobleaching. J. Microsc. *227*, 98–109. https://doi.org/10.1111/j.1365-2818.2007.01794.x.

Sjollema, K.A., and Giepmans, B.N.G. (2016). Automated annotating label in nanotomy. Imaging Microsc. *4*, 45–46.

Slot, J.W., and Geuze, H.J. (1981). Sizing of protein A-colloidal gold probes for immunoelectron microscopy. J. Cell Biol. *90*, 533–536. https://doi.org/10.1083/jcb.90.2.533.

Slot, J.W., and Geuze, H.J. (1985). A new method of preparing gold probes for multiple-labeling cytochemistry. Eur. J. Cell Biol. *38*, 87–93.

Slot, J.W., and Geuze, H.J. (2007). Cryosectioning and immunolabeling. Nat. Protoc. *2*, 2480–2491. https://doi.org/10.1038/nprot.2007.365.

Smeets, M.. METEOR: An integrated top down cryo-CLEM imaging system. https://blog.delmic.com/meteor-an-integrated-top-down-cryo-clem-imaging-system.

Stierhof, Y.D., and Schwarz, H. (1989). Labeling properties of sucrose-infiltrated cryosections. Scanning Microsc. *3*, 35–46. http://www.ncbi.nlm.nih.gov/pubmed/2694272.

Stierhof, Y.-D., Schwarz, H., and Frank, H. (1986). Transverse sectioning of plastic-embedded immunolabeled cryosections: morphology and permeability to protein A-colloidal gold complexes. J. Ultrastruct. Mol. Struct. Res. *97*, 187–196. https://doi.org/10.1016/S0889-1605(86)80017-9.

Sun, R., Liu, Y.T., Tao, C.L., Qi, L., Lau, P.M., Zhou, Z.H., and Bi, G.Q. (2019). An efficient protocol of cryo-correlative light and electron microscopy for the study of neuronal synapses. Biophys. Rep. *5*, 111–122. https://doi.org/10.1007/s41048-019-0092-4.

Takizawa, T., Powell, R.D., Hainfeld, J.F., and Robinson, J.M. (2015). FluoroNanogold: an important probe for correlative microscopy. J. Chem. Biol. *8*, 129–142. https://doi.org/10.1007/s12154-015-0145-1.

Timmermans, F.J., Liszka, B., Lenferink, A.T.M., Van Wolferen, H.A.G.M., Otto, C., et al. (2016). Integration of correlative Raman microscopy in a dualbeam FIB SEM. J. Raman Spectrosc. *47*, 956–962. https://doi.org/10.1002/jrs.4931.

van der Beek, J., de Heus, C., Liv, N., and Klumperman, J. (2022). Quantitative correlative microscopy reveals the ultrastructural distribution of endogenous endosomal proteins. J. Cell Biol. *221*. https://doi.org/10.1083/jcb.202106044.

van Driel, L.F., Valentijn, J.A., Valentijn, K.M., Koning, R.I., and Koster, A.J. (2009). Tools for correlative cryo-fluorescence microscopy and cryo-electron tomography applied to whole mitochondria in human endothelial cells. Eur. J. Cell Biol. *88*, 669–684. https://doi.org/10.1016/j.ejcb.2009.07.002.

Van E., D.M., Bos, E., Pawlak, J.B., Overkleeft, H.S., Koster, A.J., VAN Kasteren, S.I., et al. (2017). Correlative light and electron microscopy reveals discrepancy between gold and fluorescence labelling. J. Microsc. *267*, 309–317. https://doi.org/10.1111/jmi.12567.

van Rijnsoever, C., Oorschot, V., and Klumperman, J. (2008). Correlative light-electron microscopy (CLEM) combining live-cell imaging and immunolabeling of ultrathin cryosections. Nat. Methods *5*, 973–980. https://doi.org/10.1038/nmeth.1263.

Verkade, P. (2008). Moving EM: the rapid transfer system as a new tool for correlative light and electron microscopy and high throughput for high-pressure freezing. J. Microsc. *230*, 317–328. https://doi.org/10.1111/j.1365-2818.2008.01989.x.

Vicidomini, G., Gagliani, M.C., Canfora, M., Cortese, K., Frosi, F., Santangelo, C., Di Fiore, P.P., Boccacci, P., Diaspro, A., and Tacchetti, C. (2008). High data output and automated 3D correlative light-electron microscopy method. Traffic *9*, 1828–1838. https://doi.org/10.1111/j.1600-0854.2008.00815.x.

Vicidomini, G., Gagliani, M.C., Cortese, K., Krieger, J., Buescher, P., Bianchini, P., Boccacci, P., Tacchetti, C., and Diaspro, A. (2010). A novel approach for correlative light electron microscopy analysis. Microsc. Res. Tech. *73*, 215–224. https://doi.org/10.1002/jemt.20777.

Villa, E., Schaffer, M., Plitzko, J.M., and Baumeister, W. (2013). Opening windows into the cell: focused-ion-beam milling for cryo-electron tomography. Curr. Opin. Struct. Biol. *23*, 771–777. https://doi.org/10.1016/j.sbi.2013.08.006.

Wagner, F.R., Watanabe, R., Schampers, R., Singh, D., Persoon, H., Schaffer, M., Fruhstorfer, P., Plitzko, J., and Villa, E. (2020). Preparing samples from whole cells using focused-ion-beam milling for cryo-electron tomography. Nat. Protoc. *15*, 2041–2070. https://doi.org/10.1038/s41596-020-0320-x.

Watanabe, S., Punge, A., Hollopeter, G., Willig, K.I., Hobson, R.J., Davis, M.W., Hell, S.W., and Jorgensen, E.M. (2011). Protein localization in electron micrographs using fluorescence nanoscopy. Nat. Methods *8*, 80–84. https://doi.org/10.1038/nmeth.1537.

Waterhouse, A., Bertoni, M., Bienert, S., Studer, G., Tauriello, G., Gumienny, R., Heer, F.T., de Beer, T.A., Rempfer, C., Bordoli, L., et al. (2018). SWISS-MODEL: homology modelling of protein structures and complexes. Nucleic Acids Res. *46*, W296–W303. https://doi.org/10.1093/nar/gky427.

Weisenburger, S., Jing, B., Renn, A., and Sandoghdar, V. (2013). Cryogenic localization of single molecules with angstrom precision. In Nanoimaging and Nanospectroscopy, P. Verma and A. Egner, eds. (International Society for Optics and Photonics), p. 88150D. https://doi.org/10.1117/12.2025373.

Zeuschner, D., Geerts, W.J., van Donselaar, E., Humbel, B.M., Slot, J.W., Koster, A.J., and Klumperman, J. (2006). Immuno-electron tomography of ER exit sites reveals the existence of free COPII-coated transport carriers. Nat. Cell Biol. *8*, 377–383. https://doi.org/10.1038/ncb1371.

Zhang, Y., and Hensel, M. (2013). Evaluation of nanoparticles as endocytic tracers in cellular microbiology. Nanoscale *5*, 9296. https://doi.org/10.1039/c3nr01550e.

Zheng, S.Q., Palovcak, E., Armache, J.P., Verba, K.A., Cheng, Y., and Agard, D.A. (2017). MotionCor2: anisotropic correction of beam-induced motion for improved cryo-electron microscopy. Nat. Methods *14*, 331–332. https://doi.org/10.1038/nmeth.4193.

STAR★METHODS

KEY RESOURCES TABLE

REAGENT or RESOURCE	SOURCE	IDENTIFIER
**Antibodies**		
Mouse anti-LAMP1	BD Pharmigen	Cat# 555798, RRID:AB_396132
Rabbit anti-EEA1	Cell Signaling technology	Cat# 3288, RRID:AB_2096811
Mouse anti-CD63	Developmental Studies Hybridoma Bank	Cat# h5c6, RRID:AB_528158
Rabbit anti-Mouse	ZYMED	Cat# 61-6800, RRID:AB_88323
Donkey anti-Mouse Alexa-488	Life Technologies	Cat# A-21202, RRID:AB_141607
Goat anti-Mouse Alexa-647	Life Technologies	Cat# A-21235, RRID:AB_2535804
Donkey anti-Rabbit Alexa-647	Life Technologies	Cat# A-31573, RRID:AB_2536183
Donkey anti-Rabbit Alexa-568	Life Technologies	Cat# A10042, RRID:AB_2534017
Donkey anti-Rabbit Alexa-488	Life Technologies	Cat# A-21206, RRID:AB_2535792
Protein A – Au conjugate (10 nm)	Cell Microscopy Core, UMC Utrecht	https://cellbiology-utrecht.nl/products.html
**Chemicals, peptides, and recombinant proteins**		
fBSA-Au^5	Cell Microscopy Core, UMC Utrecht	https://cellbiology-utrecht.nl/products.html
fBSA-Au^{10}	Cell Microscopy Core, UMC Utrecht	https://cellbiology-utrecht.nl/products.html
Chloroauric acid trihydrate	Merck	1.01582
Tannic Acid	Mallinckrodt Pharmaceuticals	1764
Bovine Serum Albumin	Sigma-Aldrich	A9647
BSA Alexa Fluor™ 555 conjugate	Invitrogen, ThermoFisher	A34786
Protein-A	GE Healthcare	17-0872-50
Methylcellulose	Sigma-Aldrich	M-6385
Paraformaldehyde	Sigma-Aldrich	441244
Glutaraldehyde	Polysciences	1201
Osmium Tetroxide	Electron Microscopy Sciences	19132
Uranylacetate	SPI-Chem	02624-AB
Embed 812	Electron Microscopy Sciences	14900
Lowicryl HM20	Polysciences	23994
**Deposited data**		
Raw and analyzed data	This paper	N/A
**Experimental models: Cell lines**		
Human: HeLa cells	ATCC	CCL-2
Human: U2OS cells	ATCC	HTB-96
Mouse: Dorsal root ganglion (DRG) neurons	Foster et al., 2022	N/A
**Software and algorithms**		
ImageJ	Schneider et al., 2012	https://imagej.nih.gov/ij/
Icy	De Chaumont et al., 2012	https://icy.bioimageanalysis.org/
ec-CLEM	Paul-Gilloteaux et al., 2017	https://icy.bioimageanalysis.org/plugin/ec-clem/
PSA particle size analyzer for ImageJ	Ralph Sperling, Institut Català de Nanotecnologia	https://github.com/psa-rs/psa-macro
ComDet	Eugene Katrukha, Utrecht University	https://imagej.net/plugins/spots-colocalization-comdet
IMOD	Mastronarde and Held, 2017	https://bio3d.colorado.edu/imod/
serialEM	Mastronarde, 2018	https://bio3d.colorado.edu/SerialEM/index.html
**Other**		
Electron Microscopy Grids	This paper	https://cellbiology-utrecht.nl/products.html

RESOURCE AVAILABILITY

Lead contact

Information and requests for resources and reagents can be directed to and will be fulfilled by the Lead Contact, Nalan Liv, PhD. (N.Liv@umcutrecht.nl).

Materials availability

Cell lines used in this study can be requested through the Lead contact. Endocytic fBSA-Au5 and fBSA-Au10 fiducials reported here are available in the product listing of Cell Microscopy Core, UMC Utrecht.

Data and code availability

- All data reported in this paper will be shared by the lead contact upon request.
- This paper does not report original code.
- Any additional information required to reanalyze the data reported in this paper is available from the lead contact upon request.

EXPERIMENTAL MODEL AND SUBJECT DETAILS

Cell lines and culture

Hela (ATCC, CCL-2) and U2OS (ATCC, HTB-96) cells were cultured in Dulbecco's Modified Eagle's Medium (DMEM; Gibco) supplemented with 10% heat-inactivated fetal bovine serum (FBS), 2mM L-glutamine, 100 U/ml penicillin, 100 μg/mL streptomycin (complete DMEM). Cells were grown under 5% CO2/air atmosphere at 37°C.

Primary dorsal root ganglion (DRG) neuron cultures were derived from spines of 6–8 week old wild-type mice after CO2 inhalation and exsanguination as described in (Foster et al., 2021). Experiments were licensed under the UK Animals (Scientific Procedures) Act of 1986 following local ethical approval. All procedures were carried out in accordance with UK Home Office regulations. DRG from each spine were isolated and kept at 4°C in HBSS (Thermo Fisher) supplemented with 20 mM Hepes pH 7.4 ('HBSS + H'). The ganglia were washed twice by pelleting at 800g for 3 min and resuspending in 5 mL 'HBSS + H' then enzymatically digested by resuspension in 1 mL 37°C HBSS supplemented with 15 μL 20 mg/mL collagenase type IV (Thermo Fisher). After 1 h incubation at 37°C, 5% CO2, 1 mL pre-warmed HBSS supplemented with 100 μL 2.5% Trypsin (Gibco) was added. After 15 min, 5 mL 'plating media' containing Neurobasal media (Thermo Fisher), 1 x B-27 (Thermo Fisher), 2 mM L-Glutamine (Thermo Fisher), 5% FBS (Thermo Fisher), 20 mM Hepes pH 7.4, 100 U/mL penicillin and 100 U/mL streptomycin was added. Trituration was performed using a 1mL pipette after washing twice in 2 mL 'plating media'. The resulting cell suspension was layered onto a 4°C, 3 mL 15% BSA cushion (BSA in DMEM) and spun at 4°C, 300g for 8 min. The cell pellet was resuspended in 0.5 mL pre-warmed 'plating' media supplemented with 100 ng/μL NGF (Peprotech). Cells from all spines were pooled before plating in microfluidic devices (MFDs) or on cryo-EM grids. Next day, media was replaced with maintenance media containing Neurobasal media, 1 x B-27, 2 mM L-Glutamine, 20 mM Hepes pH 7.4, 100 U/mL penicillin, 100 U/mL streptomycin, 100 ng/μL NGF and 40 μM 5-UfDU (uridine and 5-fluorodeoxyuridine, Sigma). Cultures were maintained at 37°C, 5% CO2 and half media replaced every 2–3 days.

METHOD DETAILS

fBSA-Au^5 and fBSA-Au^{10} complex synthesis

5 or 10 nm colloidal gold particles were synthesized by reduction of chloroauric acid with tannic acid and sodium citrate, according to protocols developed by Slot and Geuze (Slot and Geuze (1985). In short, to make 100mL of Au particle solution, Solution A containing 80 mL H_20 + 1 mL 1% gold chloride and Solution B containing 4 mL 1% tri-sodium citrate.2H20 + 16 mL H_20 + a variable amount of 1% tannic acid (depending on the aimed Au size) were prepared, heated up to 60°C, and mixed while stirring. When the red color formed (indicative of forming Au particles), the solution was heated to 95°C and then cooled on ice. Reagent ratios were adjusted to obtain 5 or 10 nm sized colloid gold particles. Following synthesis, the colloid particles were stabilized with an excess of AlexaFluor 555-labeled BSA, as described previously for other proteins (Slot and Geuze, 1981, 1985). The pH of the Au solution was adjusted to 6 with 0. 1 N NaOH, 25 μg/mL AlexaFluor 555-labeled BSA was added while stirring the solution. For additional stabilization, 0.1% BSA (final concentration) was added to the solution. The complexes were centrifuged on a 10–30% glycerol gradient centrifugation to remove aggregates and excess protein. The purified fraction was diluted in PBS and stored with the addition of sodium azide. Prior to use in cell culture, the required volume of fBSA-Au was dialyzed against PBS overnight at 4°C to remove the sodium azide and residual contaminants.

Measurements for sizing of synthesized gold colloids were performed by diluting gold colloids or fBSA-Au at OD5 in dH_2O. Formvar and carbon-coated copper grids were placed on 5 μL drops of diluted solutions for 5 min. Grids were washed once on drops of dH_2O, after which excess liquid was drained using filter paper. After drying, the gold particles were then imaged in TEM at magnifications >80,000×.

Immunofluorescence labelling and imaging of endocytosed fBSA-Au

Cells grown on glass coverslips were treated with fBSA-Au[5] in culture medium at OD5, and incubated for 3 h at 37°C. Cells were fixed with 4% formaldehyde in PBS for 1 h, and permeabilized with 0.1% Triton X-100 in PBS. Blocking was performed using 1% bovine serum albumin (BSA) in PBS. Immunolabeling for LAMP-1 and EEA-1 was performed by incubating coverslips in PBS containing the corresponding antibodies and 1% BSA. Labeling was visualized using Alexa-tagged secondary antibodies. After secondary labeling, coverslips were washed with PBS and dH_2O, and mounted to microscope slides using Prolong Gold or Diamond (Thermo Scientific).

Cells were imaged as z -stacks in a Deltavision RT widefield FM (GE Healthcare, U.S.A.), using 100 ×1.4-NA oil objective lens. The microscope was equipped with a Cascade II EM-CCD camera (Photometrics, U.S.A.), and a gain value of 40 was used. Images were acquired using the Acquire3D module in Softworx 6.5.2.

Correlative microscopy of resin-embedded samples

For correlation of fluorescence microscopy and EM of resin-embedded cells, imaging was performed prior to sample preparation in EM. Cells were grown on carbon-coated, gridded coverslips prepared as in (Fermie et al., 2018), and treated with fBSA-Au[5] diluted to OD5 in complete DMEM for 3 h. Cells were washed in 1× PHEM buffer to remove excess fBSA-Au[5], and fixed using 4% formaldehyde and 0.2% glutaraldehyde in 1× PHEM buffer. Using FM, Z-stacks of cells of interest were obtained for the Alexa 555 signal. The position of cells relative to the pattern etched in the coverslip was registered using polarized light.

To prepare specimens for electron microscopy, the imaged coverslips were postfixed using osmium tetroxide and uranyl acetate, dehydrated using a graded ethanol series, and embedded in Epon resin. Resin was polymerized for 48 h at 65°C. After polymerization, the glass coverslip was removed from the Epon block by dissolving it in hydrogen fluoride, after which the exposed Epon surface was thoroughly cleaned with distilled water and left to harden overnight at 63°C. Areas of the resin block containing imaged cells were cut out using a clean razor blade, and glued to empty Epon sample stubs, with the basal side of the cells facing outwards. From these blocks, 70 and 250 nm thick sections were cut and collected on formvar and carbon coated copper support grids (50 mesh or slot grids). Grids with 250 nm thick sections were seeded with tomography fiducials by placing the grids on drops of ddH_2O containing 1:100 diluted protein-A-gold 10 nm for 5 min. Afterwards, grids were rinsed 3 times on distilled water and blotted dry with filter paper.

For correlation of HM20 embedded samples, HeLa cells were grown in 6cm petri-dishes, and incubated with fBSA-Au[5] diluted to OD5 in complete DMEM for 3 h. The cells were scraped, high-pressure frozen (EM ICE, Leica Microsystems), and freeze substituted (AFS2, Leica Microsystems) in HM20 using previously described protocols to retain fluorescent signal in the samples (Peddie et al., 2014). Then sections of 100nm were prepared on TEM grids. The sections were first imaged in the Deltavision RT widefield FM (GE Healthcare, U.S.A.), and then in a Tecnai 12 TEM (Thermo Fischer Scientific, Eindhoven, The Netherlands).

Sample preparation and light microscopic imaging of Tokuyasu cryosections

For CLEM on thin (70 nm) and thick (350 nm) cryosections, cells were grown in 60 mm culture dishes, treated with fBSA-Au[5] diluted to OD5 in complete DMEM for 3 h at 37°C and fixed with 2% formaldehyde and 0.2% glutaraldehyde in 0.1M phosphate buffer (pH 7.4). Samples were gelatin embedded, cryoprotected, sectioned and immunolabeled according to previous protocols (Slot and Geuze, 2007; van Rijnsoever et al., 2008), with minor modifications. Following incubation with primary antibodies, the grids were labeled with Alexa 488 labeled secondary antibodies, followed by incubation with protein-A gold conjugates (10 nm). The grids were washed with dH_2O and placed between a microscope slide and a #1 coverslip in 2% methylcellulose in dH_2O. Sections were imaged in a Deltavision RT widefield FM (GE Healthcare, U.S.A.) equipped with a Cascade II EM-CCD camera (Photometrics, U.S.A.). Grids were first imaged at 40× magnification to form a map of the section, after which regions of interest were selected using 100× magnification. After imaging the grids were removed from the microscope slide, thoroughly rinsed with H_2O and contrasted for EM and embedded in methylcellulose containing uranyl acetate, according to previous protocol (Slot and Geuze, 2007).

Electron microscopy of resin sections and Tokuyasu cryosections

Thin cryosections were imaged in a Tecnai 12 TEM (Thermo Fischer Scientific, Eindhoven, The Netherlands) equipped with a Veleta 2k×2k CCD camera (EMSIS, Munster, Germany), operating at 80 kV. Tilt series of resin sections and labeled thick cryosections were acquired in a Tecnai 20 TEM (Thermo Fischer Scientific) operating at 200 kV, equipped with an Eagle 4K×4K CCD camera running Xplore3D (Thermo Fischer Scientific) software. Single tilt image series were automatically collected with 1° tilt increments from −60° to +60° at microscope magnifications of 11500× or 14500×, resulting in final pixel sizes of 0.96 nm or 0.76 nm, respectively.

Cryo-FM and Cryo-EM of DRG neurons

For preparation of EM grids, a thin layer of homemade continuous carbon was floated on top of individual Quantifoil R3.5/1 200 mesh gold grids (Quantifoil Micro Tools). After drying, these were plasma cleaned using Nano Clean Plasma cleaner Model 1070 (Fischione) for 40 s at 70% power in a 9:1 mixture of Argon and Oxygen gas. Grids were transferred into a Ibidi μ-slide 2 well co-culture dish (Ibidi) and coated with poly-L-lysine and laminin as described for the MFDs. After growth of DRG for 4 DIV, fBSA-Au[5] was prepared as for live imaging and incubated for 2 h. Samples were washed twice in pre-warmed maintenance media lacking 5-UfDU then vitrified by plunge freezing into liquid ethane after manual back-side blotting in a Vitrobot Mk II (Thermo Fisher) kept at 37°C, 100% humidity.

Cryo fluorescence microscopy was performed using a Leica EM cryo-CLEM wide-field microscope (Leica Micosystems) equipped with a 50x/0.90 NA DRY cryo-objective lens. 15 μm z-stacks of the entire grid with 1 μm z-spacing were acquired using the Leica LAS

X Matrix software in green, red and transmitted light channels. Correlation was performed manually during cryo-EM imaging. Cryo-electron tomograms of the thin parts of DRG axons were acquired using a TITAN Krios G3 (Thermo Fisher) operated at 300kV equipped with K3 detector and Quantum GIF (Gatan) with slit width 20 eV. Tilt series were acquired using SerialEM (Mastronarde, 2005) from ±60° in 2° increments using a dose-symmetric scheme (Hagen et al., 2017) with defocus set from 3.5–6 μm underfocus. The dose in each image was $2e^-/Å^2$ with pixel size 2.68Å/pix, leading to total dose $120e^-/Å^2$. Movies were acquired in counting mode, with 10 frames per tilt image.

Cryo-FM and Cryo-EM of U2OS cells

Gold grids (Quantifoil, R2/2) were glow-discharged and incubated on top of 40 μL droplets of fibronectin (50 μg/mL) for 2–3 h at 37°C. 90.000 U2OS cells were seeded on these grids in 30 cm glass bottom dishes (Greiner bio-one). After 72 h, 3 μL of 1 μm Dynabeads (Thermo Fischer Scientific: MyOne with 40% iron oxide, carboxylic acid) diluted 1:10 in PBS, was added to the grids and the cells were vitrified in liquid ethane after manually blotting for 12s. Prior to plunging, the cells were incubated at 37°C with fBSA-Au^5 diluted to OD 5 forOD5 for 3 to 4 h.

fBSA-Au^5 spots particles were localized in the FEI CorrSight^{TM}, a spinning-disk confocal microscope equipped with a cryo-module. Grid overview images were taken with a 5x/0.16 NA air objective. Individual cells were imaged with a 40x/0.9 NA air objective as 10 μm deep z -stacks with 300 nm spacing, after excitation at 488 (Dynabeads) and 561 (fBSA-Au^5) nm. Image acquisition was done using FEI MAPS 3.8 software. Next, the grid was loaded in the cryo-FIB (Aquilos^{TM}, Thermo Fisher Scientific), the SEM grid overview image was correlated to the light microscope overview image in MAPS3.8 using the 3-point alignment method. Individual z-stacks were correlated to the SEM image (1536x1024, 1 μs, 2 kV, 13 pA) of target cells, prior and post milling. The Dynabeads were used as fiducials to perform the x-y-z correlation between the SEM images and the z-stacks. Transformation parameters were determined using the 3D Correlation Toolbox and the transform was applied using Pyto, a python-based package for cryo-ET analysis, as described in Arnold et al. (2016).

Lamellae were prepared as described in Wagner et al. (2020), at 16 degree stage tilt with a stepwise decreasing current of 1 to 0.3 to 0.1 nA. The final polishing step was performed at 30 pA to reach a lamella thickness of 100–200 nm. Lamellae were imaged on a 200 kV Talos Arctica transmission electron microscope (Thermo Fisher Scientific) with a post-column energy filter (slit width of 20 eV) and a K2 summit direct electron detector (Gatan). Lamellae overview images were recorded at a pixel size of 18.72 Å/px. The lamellae visible in the previously recorded post-milling SEM images were overlayed in FIJI with the lamella overview images recorded in the cryo-TEM to allow correlation of the LM data to the cryo-TEM lamella overview images and thus the localization of the beads in the lamellae. High magnification tilt series of the beads were recorded using SerialEM at a pixel size of 2.17 Å/px, a dose rate of ~3 e^-/px/s and a total dose of 100.3 $e^-/Å^2$. The tilt series were collected with a 2° tilt increment from +69° to −51° at a defocus of 3.

Tomogram reconstruction

Tomogram reconstruction was performed using the IMOD software package (Kremer et al., 1996). Tilt series from ET were aligned using bead tracking of the 10 nm gold particles used for immunolabeling (cryosections) or seeded gold fiducials (resin sections). Tomograms were generated from the aligned data using weighted back projection. For DRG neurons, gain correction, CTF-estimation and motion correction were performed using the IMOD programmes alignframes and ctfplotter. Fiducial model generation, tomogram alignment and reconstruction was performed in eTomo. The resulting volumes were binned by 4 and filtered for visualization using the deconvolution filter implemented in WARP. The tilt series from U2OS cells were aligned and dose-weighted using MotionCor2 (Zheng et al., 2017). Alignment of the tilt series via either fiducial or patch tracking, and tomogram reconstructions were performed in eTomo, part of the IMOD/4.10.29 package (Kremer et al., 1996). After CTF correction was performed in IMOD, the tomograms were reconstructed using weighted back projection and a SIRT-like filter. Tomograms were 4x-binned and low pass filtered to 40 Å for visualization using the TOM Toolbox (Nickell et al., 2005).

Correlation of light and electron microscopy images

Registration of thin section fluorescence and EM data was performed using ec-CLEM (Paul-Gilloteaux et al., 2017). Here, multiple corresponding pairs of fluorescent spots and gold particles were manually selected, after which the software automatically applies the correct scaling and transformation steps and generates overlays of FM and EM data. We used only linear transformation options to achieve the overlays shown in the Figures.

For correlation of FM and ET data, registration was first performed using ec-CLEM by overlaying FM data over a regular TEM image of the ROI, collected before the start of tilt imaging. The transformed fluorescence images were then overlaid with the tomogram slices corresponding to the region of the regular TEM image.

QUANTIFICATION AND STATISTICAL ANALYSIS

Size distribution analysis of fBSA-Au^5 and fBSA-Au^{10}

Micrographs containing gold particles were analyzed using the particle size analyzer developed by Ralph Sperling (https://github.com/psa-rs/psa-macro). Respectively 154, 176, 180, and 220 gold particles, from 3 independent preparations, were measured

per Au^{5}, fBSA-Au^{5} ,Au^{10}, and fBSA-Au^{10} size determination. These data are represented in a beeswarm plot (Figure 1H). To analyze any clustering or agglomeration in solution, size measurements of synthesized gold colloids before and after BSA stabilization were done using a dynamic light scattering instrument (Zetasizer Nano ZS, Malvern, UK). The size distribution results were analyzed and plotted in Excel.

Colocalization analysis

Images acquired using Softworx 6.5.2 software were analyzed in Fiji as maximum intensity projections. Line profiles were measured and plotted using the line segment and plot profile functions in Fiji. Colocalization of fBSA-Au with LAMP-1 and EEA1 were analyzed using the ComDet 5.5 plugin (Eugene Katrukha, Cell Biology, Utrecht University) and a custom batching macro. For the colocalization analysis resulting in Figure 1D, 102 cells from 3 independent replicates were analyzed. For Figures 1E–1G, cells from 2 independent replicates were analyzed. The percentage of colocalized particles was calculated per cell, and plotted as a beeswarm boxplot.

Correlation accuracy of FM and EM images

Accuracy of correlation between FM and EM images were analysed using the inbuilt ‘‘Show predicted error in positions’’ and ‘‘Compute the whole predicted error map’’ functions of the ec-CLEM plugin in Icy (De Chaumont et al., 2012; Paul-Gilloteaux et al., 2017).

Cell Reports Methods, Volume *2*

Supplemental information

Bimodal endocytic probe for three-dimensional correlative light and electron microscopy

Job Fermie, Leanne de Jager, Helen E. Foster, Tineke Veenendaal, Cecilia de Heus, Suzanne van Dijk, Corlinda ten Brink, Viola Oorschot, Lin Yang, Wei Li, Wally H. Müller, Stuart Howes, Andrew P. Carter, Friedrich Förster, George Posthuma, Hans C. Gerritsen, Judith Klumperman, and Nalan Liv

Supplemental Figures

Bimodal Endocytic Probe for Three-Dimensional Correlative Light and Electron Microscopy

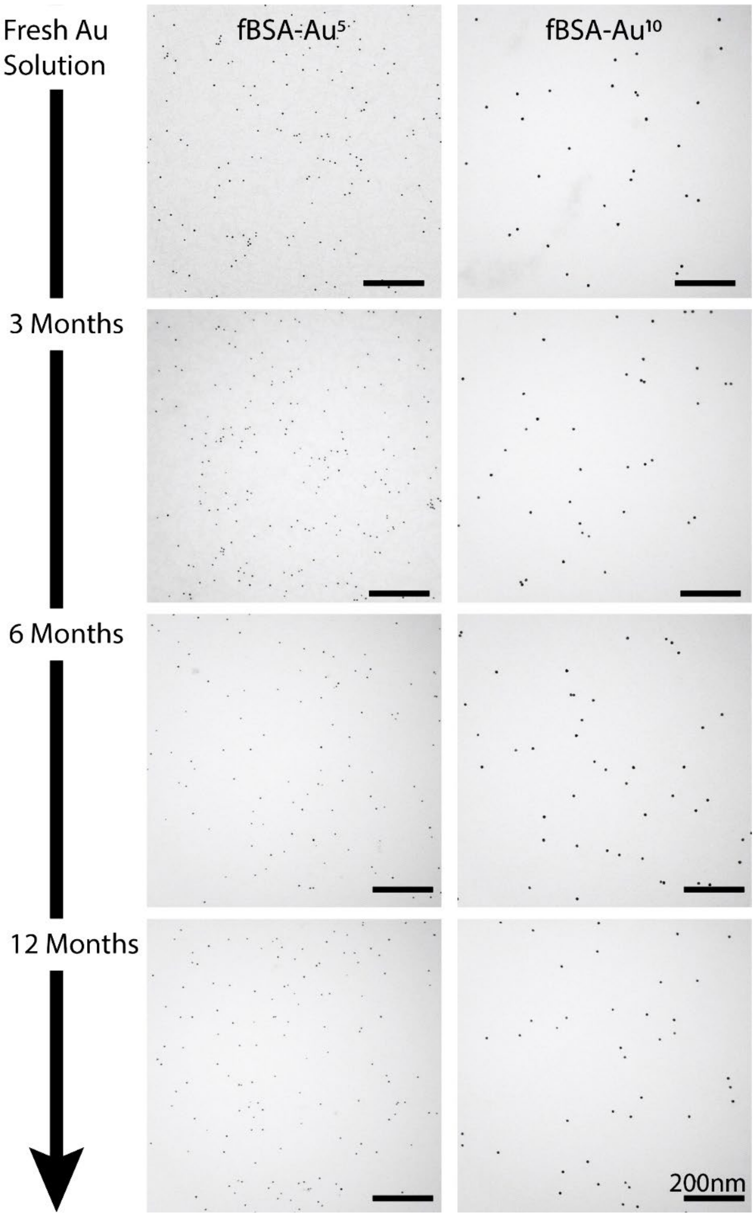

Supplementary Figure 1: fBSA-Au^{5} and fBSA-Au^{10} conjugates are stable in solution for 12 months. Related to Figure 1. TEM images of fBSA-Au^{5} (left column) and fBSA-Au^{10} (right column) particles deposited on TEM grids right after synthesis (Row 1), 3 months after synthesis (Row 2), 6 months after synthesis (Row 3), and 12 months after synthesis (Row 4). Note that even after 12 months there is no change in the size of the particles, and they remain dispersed with no clustering. *Scalebars: 200 nm*.

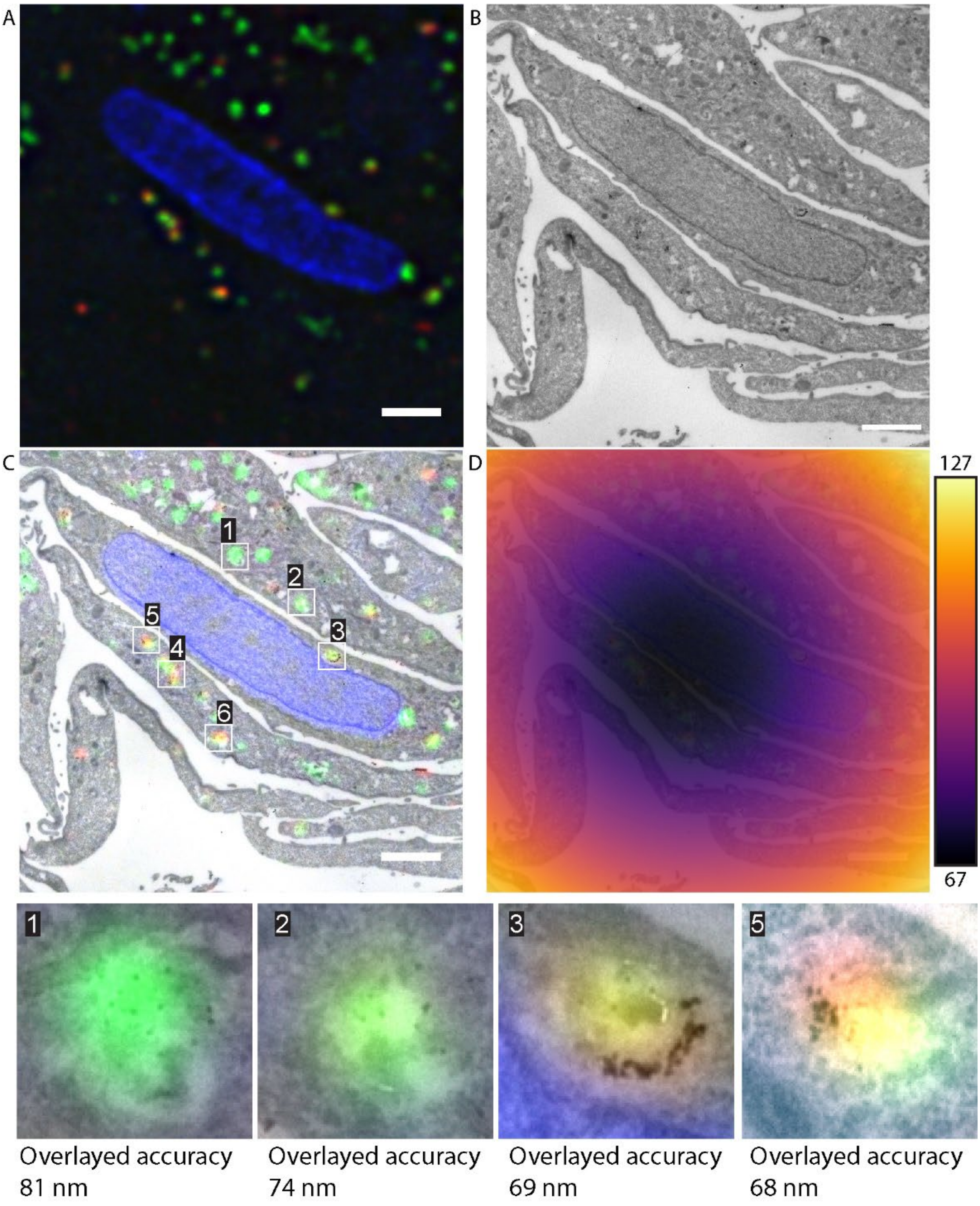

Supplementary Figure 2: fBSA-Au as fiducial enables high registration accuracy. Related to Figure 3. **(A)** FM data of region of interest on a ultrathin thawed cryosection prepared from cells with endocytosed fBSA-Au5 and immunolabelled for CD63 with Alexa488 and Au[10]. **(B)** EM region of interest. **(C)** Overlay of FM and EM data. **(D)** Quantification of registration error using ec-CLEM, showing regions of high accuracy (67 nm registration error) and lower accuracy (127 nm registration error). *Scalebars A, B, C, D: 2 µm.*

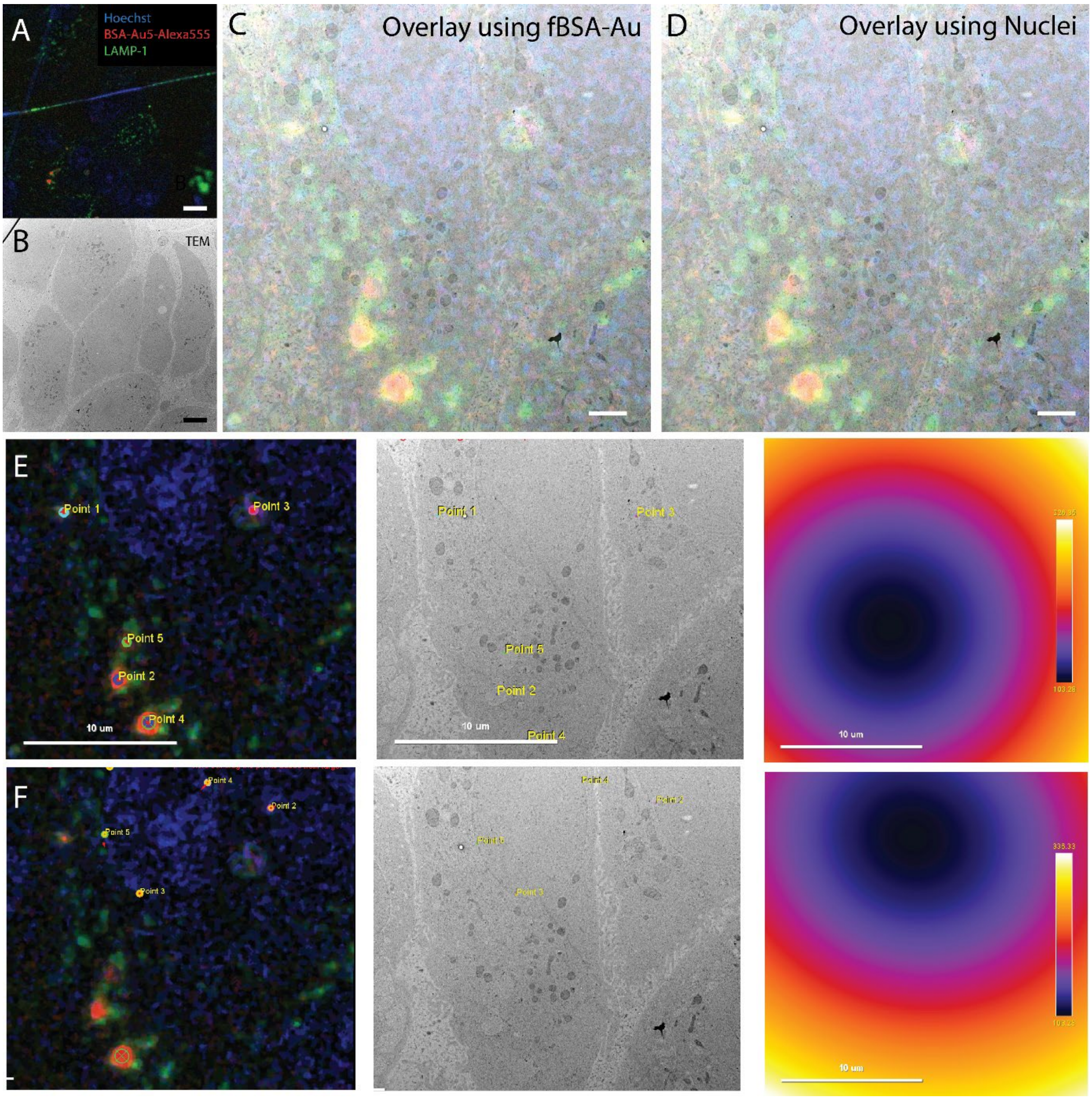

Supplementary Figure 3: Using fBSA-Au enables higher registration accuracy than using nuclei as a fiducial. Related to Figure3 and 4. (A) FM data of a region of interest on 100nm HM20 section prepared from cells with endocytosed fBSA-Au^5 and expressing LAMP-1-GFP. **(B)** EM image of the same region of interest. **(C)** Correlation of FM and EM data using five fBSA-Au^5 points in ec-CLEM software. **(D)** Correlation of FM and EM data using five nuclei edge points in and Hoechst signal in ec-CLEM software **(E)** Quantification of registration error in (C) using ec-CLEM, showing the accuracy ranges between 103 nm to 226nm. **(F)** Quantification of registration error in (D) using ec-CLEM, showing the accuracy ranges between 103 nm to 336nm. *Scalebars: (A)(B) 10 µm, (C)(D) 2 µm.*

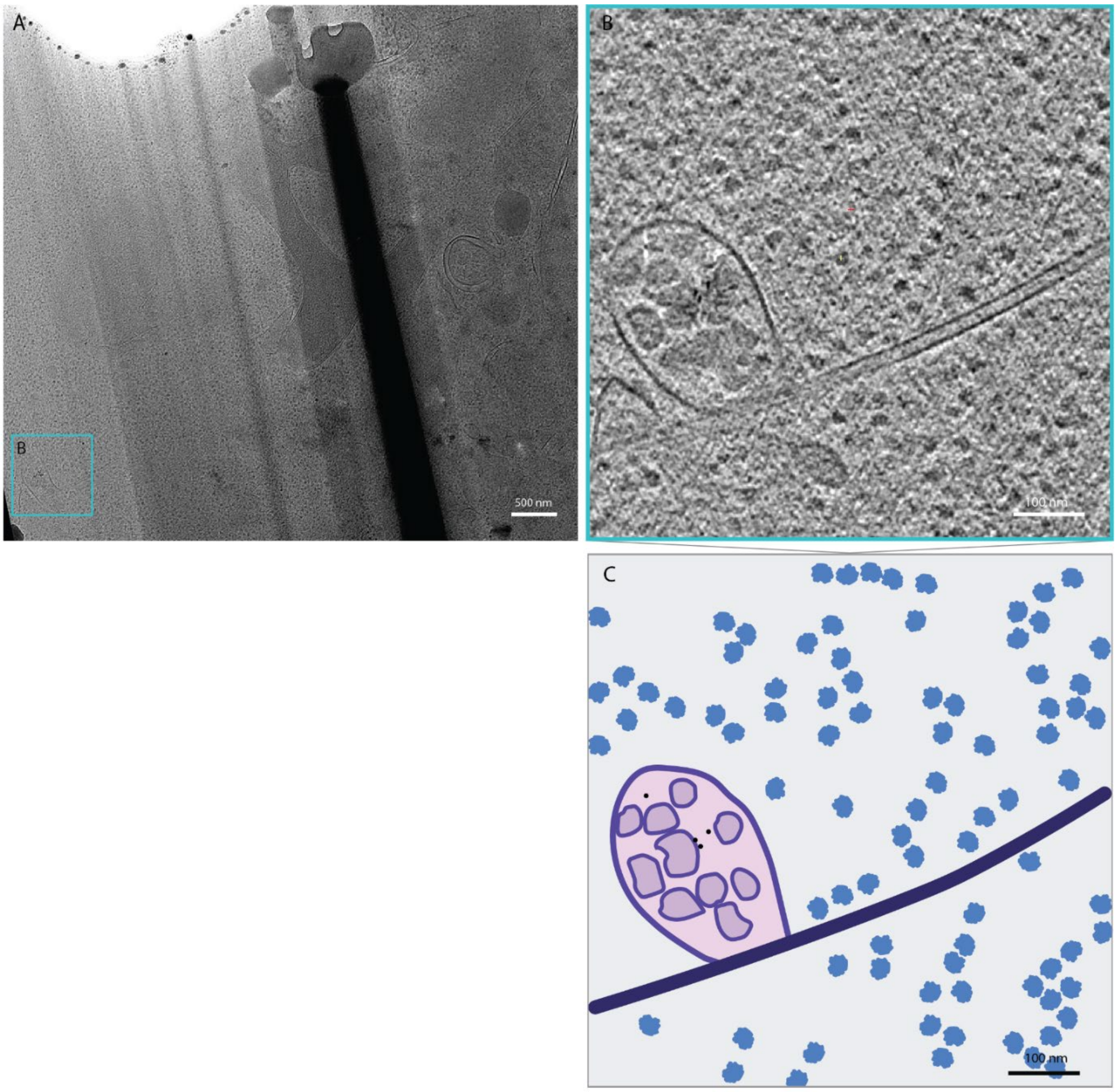

Supplementary Figure 4: Reconstruction of an endosomes containing fBSA-Au^5. Related to Figure 7. **(A)** Overview cryo-TEM image of a lamella prepared by cryo-FIB-SEM. The region imaged with higher resolution is depicted with a blue square; and shown in (B). **(B)** An endosome bearing fBSA-Au^5 in its lumen. The contact site of the endosome with the microtubule cytoskeleton is visualized in cryo-ET. **(C)** Model for the endosome-microtubule interaction with lipid bilayer shown in purple, lumen in light pink, microtubule in dark blue, ribosomes in light blue and fBSA-Au^5 shown as black circles. *Scale bars: A: 500 nm; B,C: 100 nm.*
